# Supplementary material for: Experimentally validating sabatier plot by molecular level microenvironment customization for oxygen electroreduction
Source: Nat Commun. 2024 Jul 19;15:6077. doi: 10.1038/s41467-024-50377-y (PMC11271610; doi:10.1038/s41467-024-50377-y)
Supplement: Supplementary file 1 — Supplementary Information [file 41467_2024_50377_MOESM1_ESM.pdf]

Experimentally Validating Sabatier Plot by Molecular Level Microenvironment Customization for Oxygen Electroreduction

Bingyu Huang[1,2], Qiao Gu[1], Xiannong Tang[1], Dirk Lützenkirchen-Hecht[3], Kai Yuan[1]*, and Yiwang Chen[1,2]*

[1]College of Chemistry and Chemical Engineering/Film Energy Chemistry for Jiangxi Provincial Key Laboratory (FEC), Nanchang University, Nanchang 330031, PR China. E-mail: kai.yuan@ncu.edu.cn (K. Yuan); ywchen@ncu.edu.cn (Y. Chen)
[2]College of Chemistry and Materials/Key Lab of Fluorine and Silicon for Energy Materials and Chemistry of Ministry of Education, Jiangxi Normal University, Nanchang 330022, China
[3]Faculty of Mathematics and Natural Sciences-Physics Department, Bergische Universität Wuppertal, Gauss-Str. 20, D-42119, Wuppertal, Germany

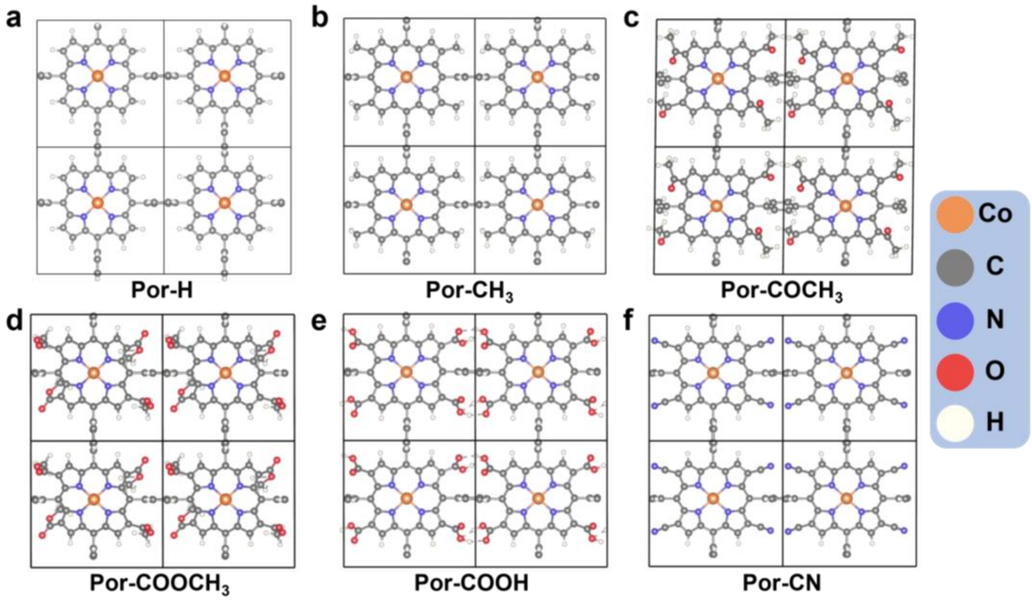

Supplementary Fig. 1. Co porphyrin-based polymer models after DFT optimization. The detailed model structures of **a** Por-H, **b** Por-CH_3, **c** Por-$COCH_3$, **d** Por-$COOCH_3$, **e** Por-COOH and **f** Por-CN.

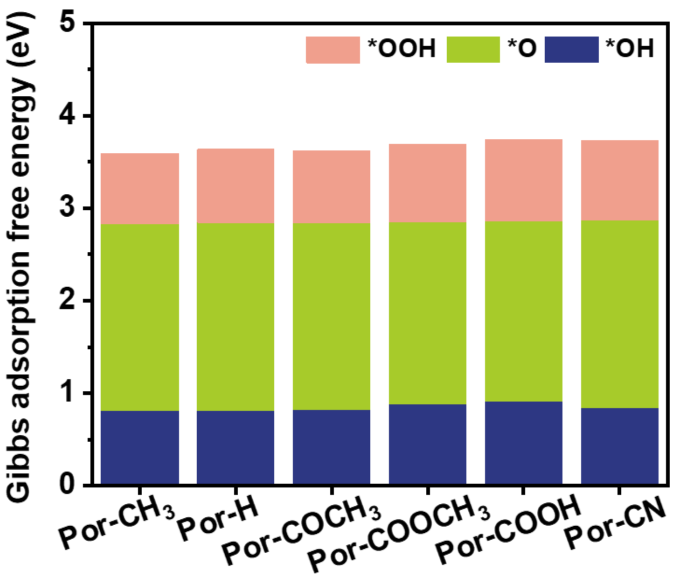

Supplementary Fig. 2. The Gibbs adsorption free energy estimation of intermediates on Co porphyrin-based polymer models. The Gibbs adsorption free energies of *OOH, *O and *OH intermediates on Por-CH_3, Por-H, Por-$COCH_3$, Por-$COOCH_3$, Por-COOH and Por-CN.

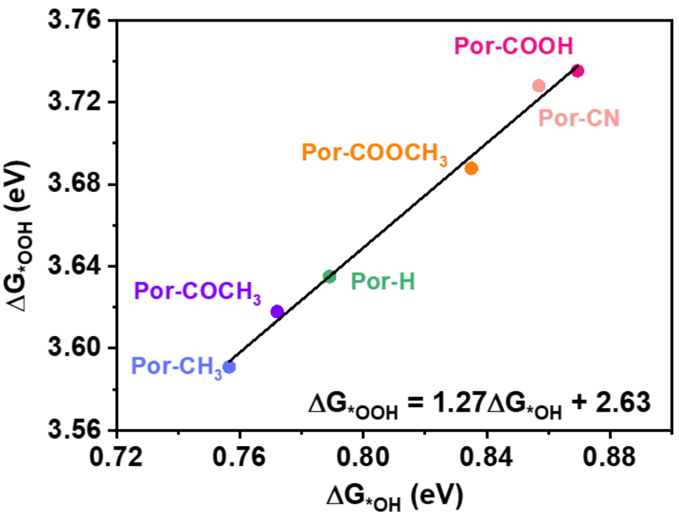

Supplementary Fig. 3. Linear relationship between ΔG_{*OOH} and ΔG_{*OH}.

The linear relationship between Gibbs free energies of *OOH intermediate (ΔG_{*OOH}) and *OH intermediate (ΔG_{*OH}) is calculated as follows:

$$\Delta G_{*OOH} = 1.27\Delta G_{*OH} + 2.63$$

The oxygen electroreduction overpotential (η) can be obtained by the following equation:

$$\eta = \max\{\Delta G_{*OOH} - 4.92, \Delta G_{*O} - \Delta G_{*OOH}, \Delta G_{*OH} - \Delta G_{*O}, -\Delta G_{*OH}\}/e + 1.23$$

Based on the abovementioned linear relationship between ΔG_{*OOH} and ΔG_{*OH}, the above equation can be transformed to:

$$\eta = \max\{1.27\Delta G_{*OH} - 2.29, \Delta G_{*O} - 1.27\Delta G_{*OH} - 2.63, \Delta G_{*OH} - \Delta G_{*O}, -\Delta G_{*OH}\}/e + 1.23$$

Thereby, theoretical η can be related to only two Gibbs free energies descriptors: ΔG_{*OH} and $\Delta G_{*O} - \Delta G_{*OH}$.

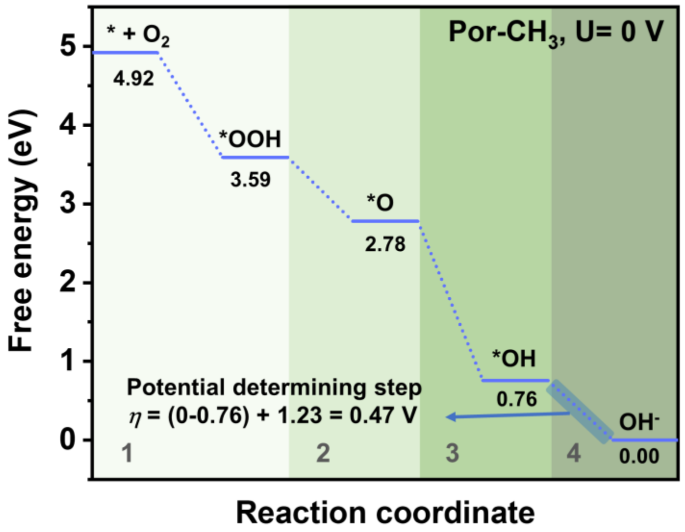

Supplementary Fig. 4. Calculated free energy diagram for ORR. The free energy diagram with four essential steps of Por-CH_3 model at U = 0 V.

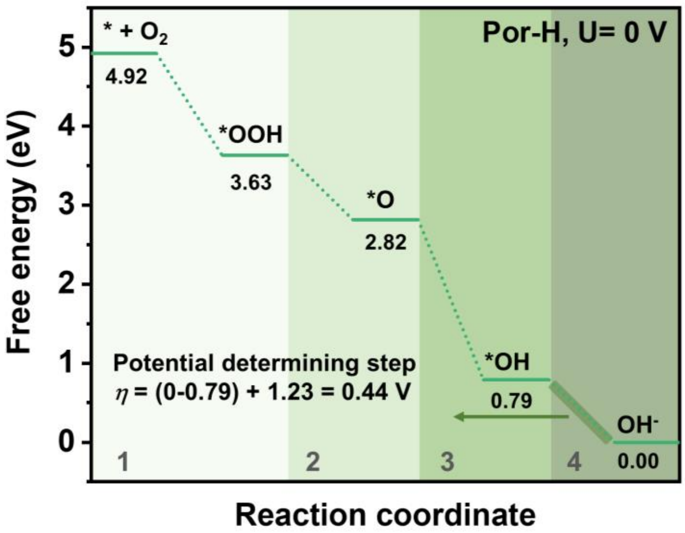

Supplementary Fig. 5. Calculated free energy diagram for ORR. The free energy diagram with four essential steps of Por-H model at U = 0 V.

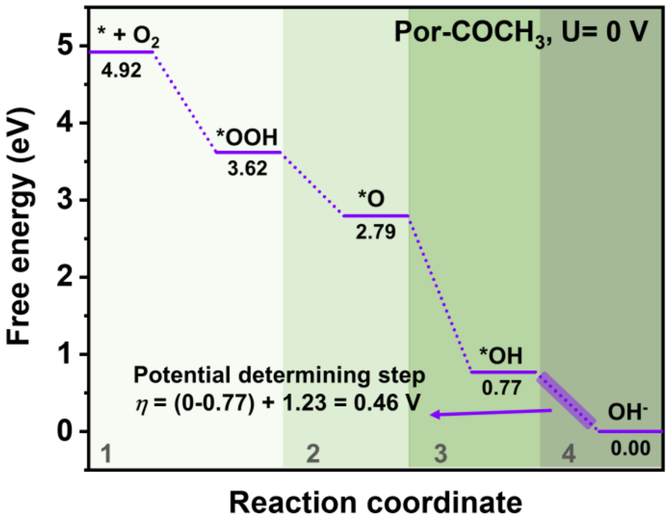

Supplementary Fig. 6. Calculated free energy diagram for ORR. The free energy diagram with four essential steps of Por-$COCH_3$ model at U = 0 V.

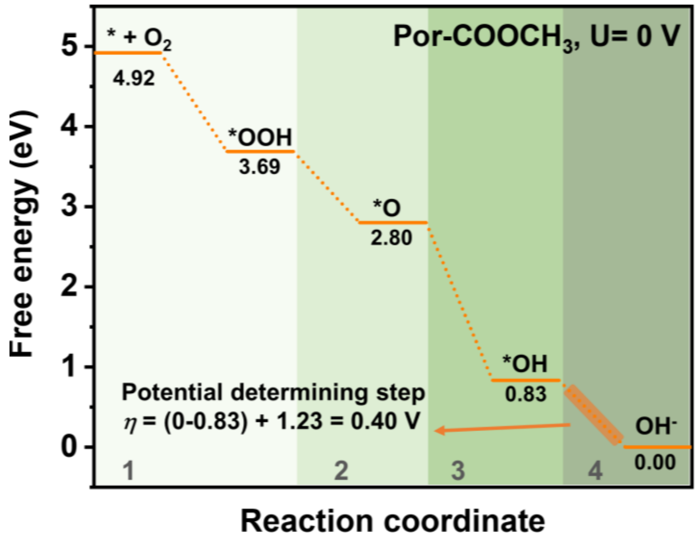

Supplementary Fig. 7. Calculated free energy diagram for ORR. The free energy diagram with four essential steps of Por-$COOCH_3$ model at U = 0 V.

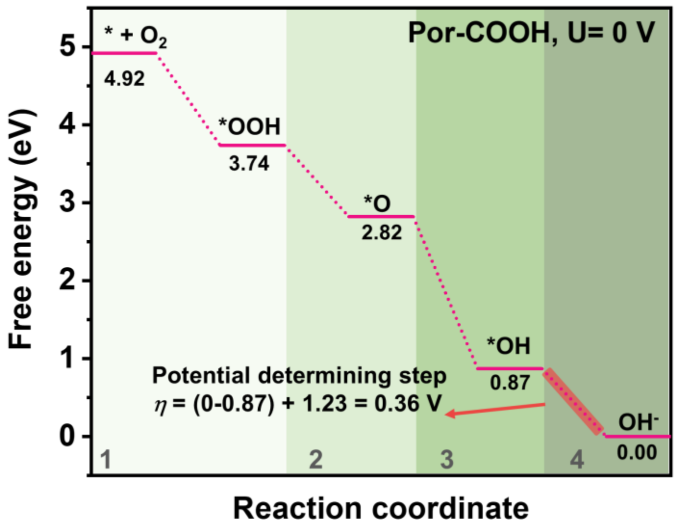

Supplementary Fig. 8. Calculated free energy diagram for ORR. The free energy diagram with four essential steps of Por-COOH model at U = 0 V.

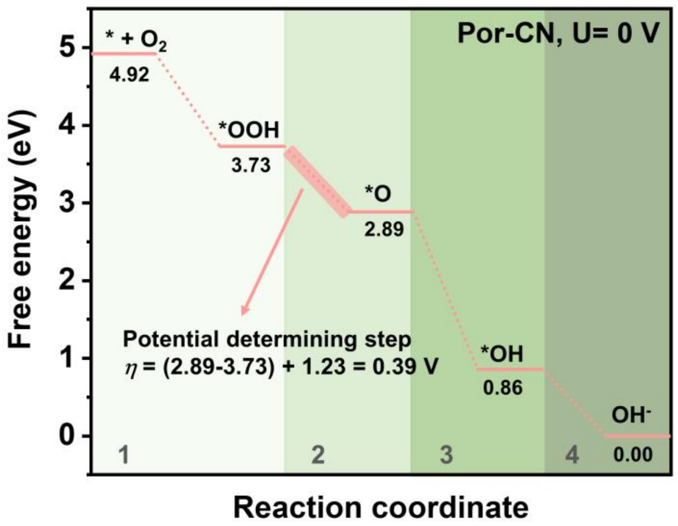

Supplementary Fig. 9. Calculated free energy diagram for ORR. The free energy diagram with four essential steps of Por-CN model at U = 0 V.

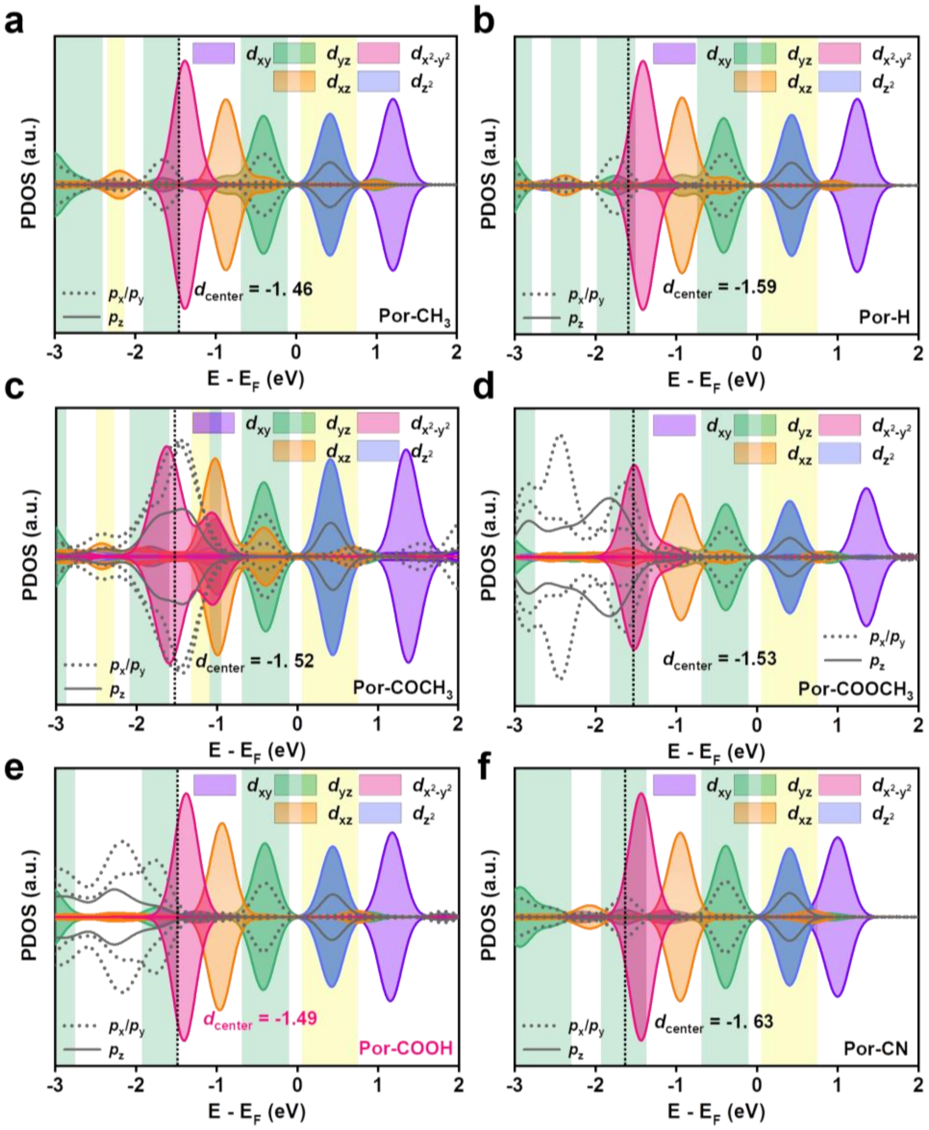

Supplementary Fig. 10. PDOS for Co 3*d* orbitals of Co porphyrin-based polymer. PDOS of *OH on **a** Por-CH_3, **b** Por-H, **c** Por-$COCH_3$, **d** Por-$COOCH_3$, **e** Por-COOH and **f** Por-CN. Yellow or green region represents the orbital overlap between p_x/p_y or p_z orbitals of *OH intermediate and d_{xz}/d_{yz} or d_{z^2} orbitals of Co atom, respectively.

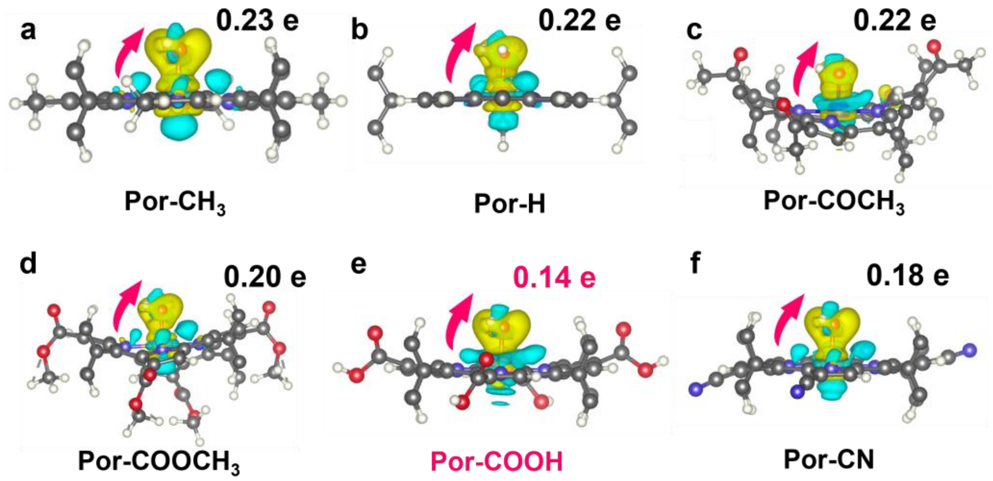

Supplementary Fig. 11. Charge density difference analysis on Co porphyrin-based polymer. The charge density differences of *OH intermediate chemisorbed on **a** Por-CH_3, **b** Por-H, **c** Por-$COCH_3$, **d** Por-$COOCH_3$, **e** Por-COOH and **f** Por-CN. Cyan and yellow represent positive and negative charges, respectively.

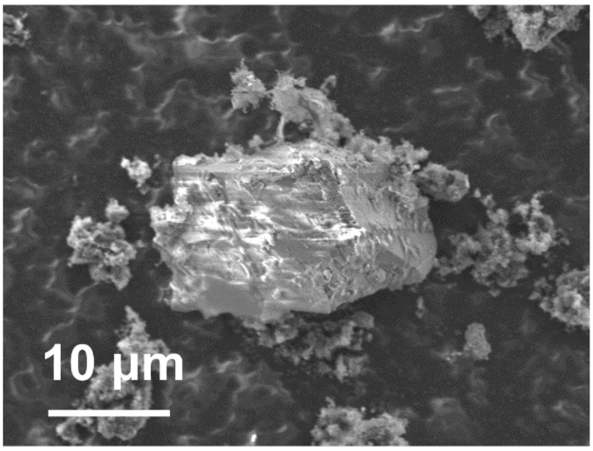

Supplementary Fig. 12. Morphology characterization. The SEM image of CoCOP-COOH.

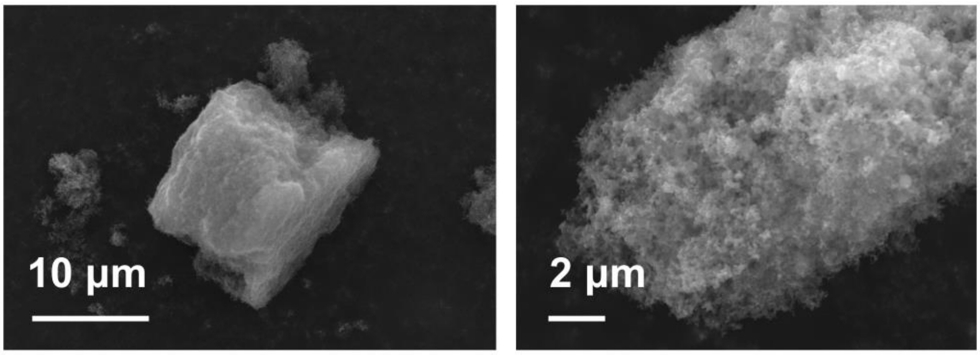

Supplementary Fig. 13. Morphology characterization. The SEM image of KB.

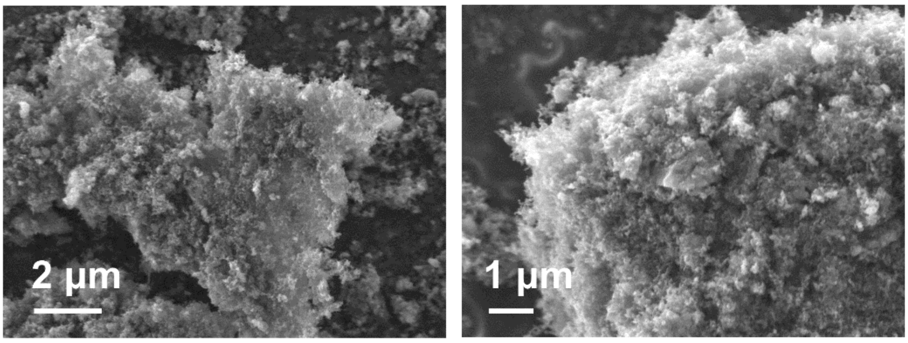

Supplementary Fig. 14. Morphology characterization. The SEM image of CoCOP-COOH@KB.

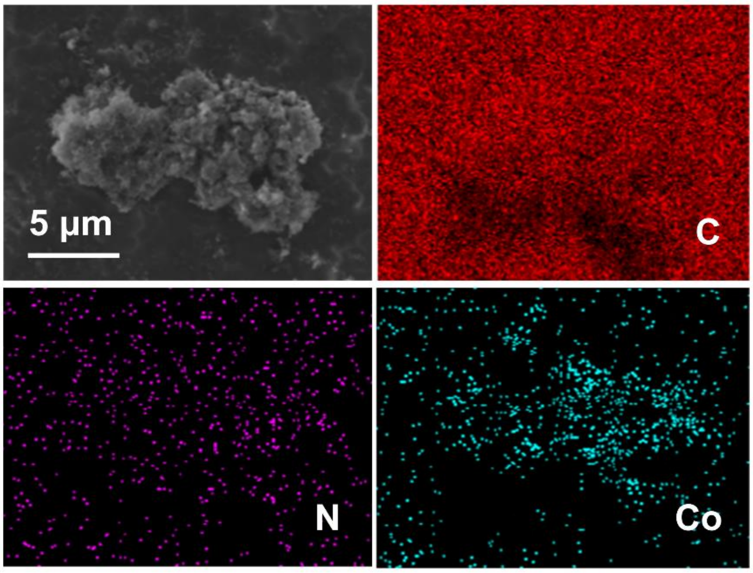

Supplementary Fig. 15. Morphology characterization. The SEM image and corresponding elemental mapping images of CoCOP-CH_3@KB.

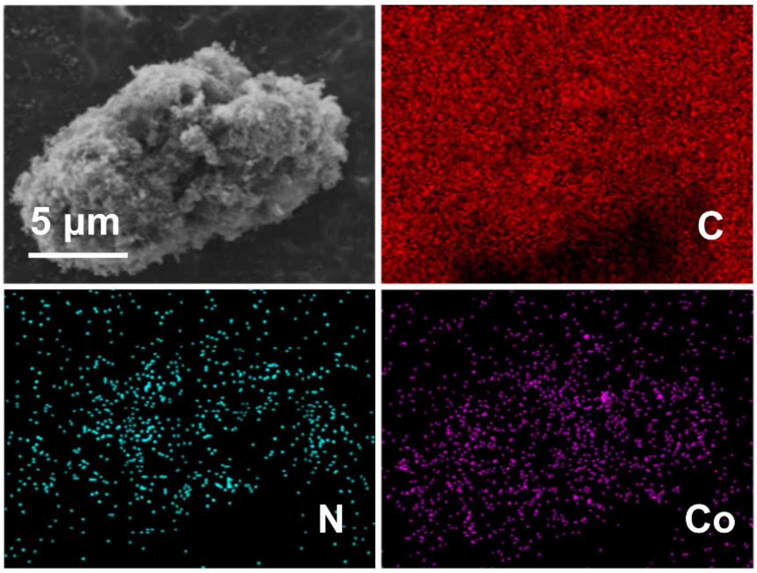

Supplementary Fig. 16. Morphology characterization. The SEM image and corresponding elemental mapping images of CoCOP-H@KB.

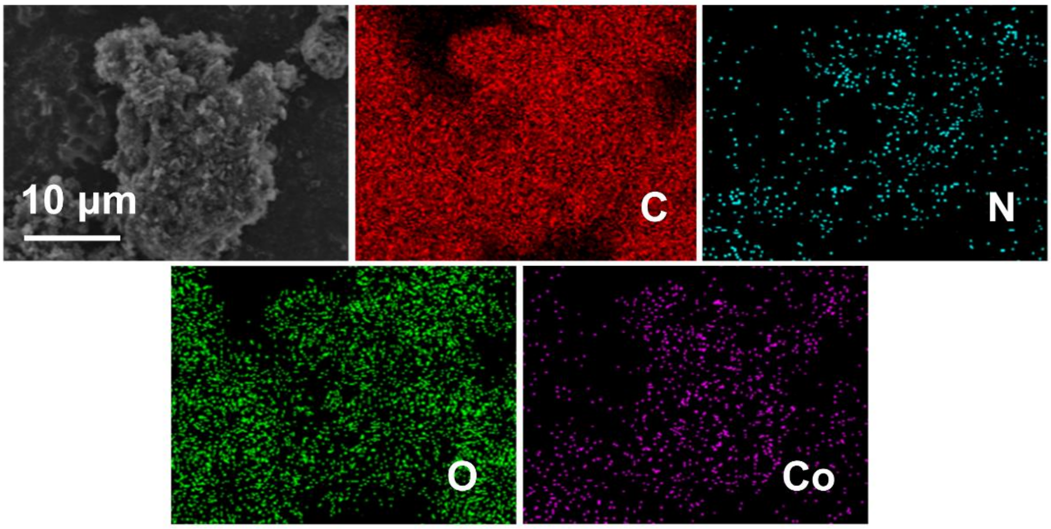

Supplementary Fig. 17. Morphology characterization. The SEM image and corresponding elemental mapping images of CoCOP-$COCH_3$@KB.

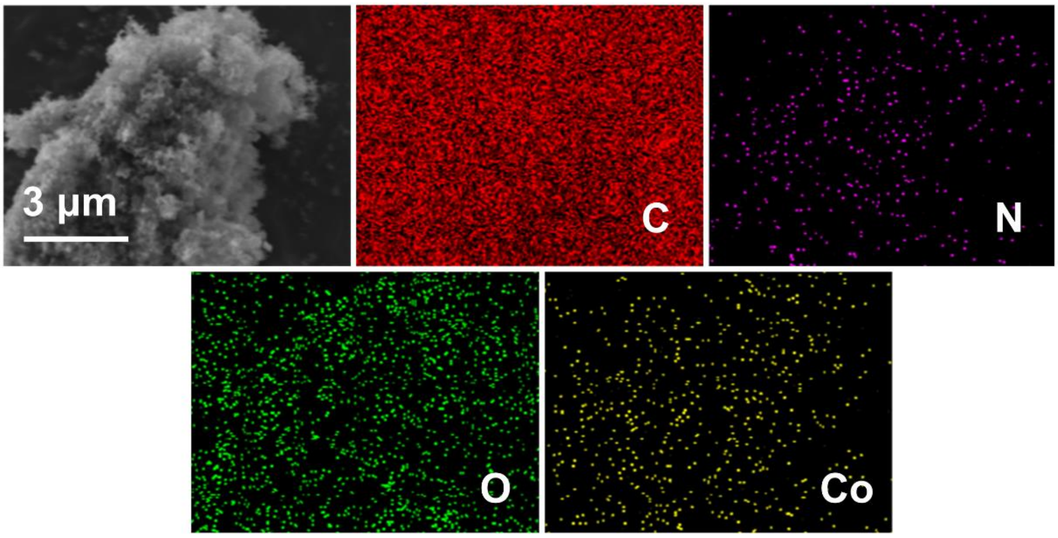

Supplementary Fig. 18. Morphology characterization. The SEM image and corresponding elemental mapping images of CoCOP-$COOCH_3$@KB.

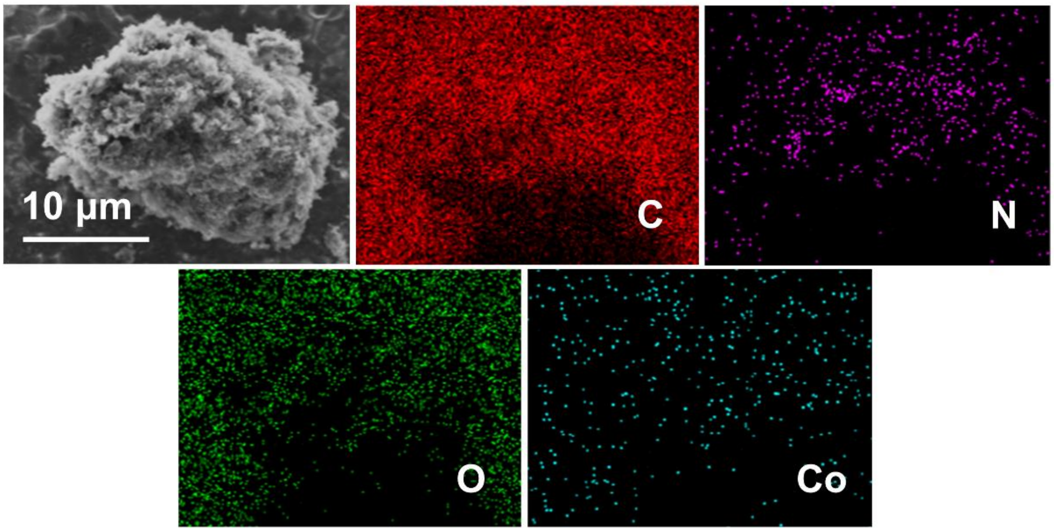

Supplementary Fig. 19. Morphology characterization. The SEM image and corresponding elemental mapping images of CoCOP-CN@KB.

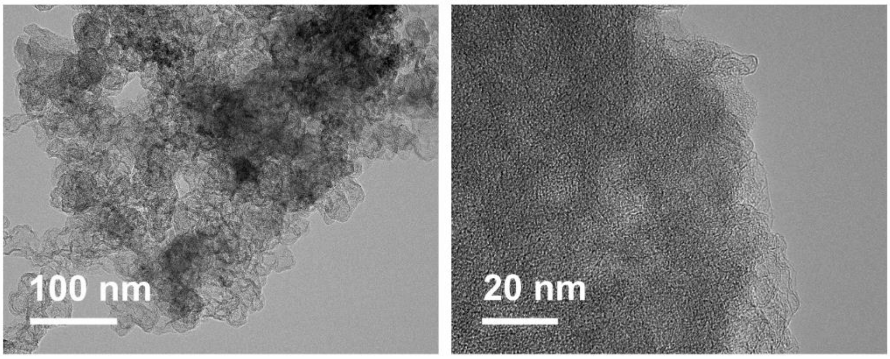

Supplementary Fig. 20. Morphology characterization. The TEM images with different magnifications of CoCOP-COOH@KB.

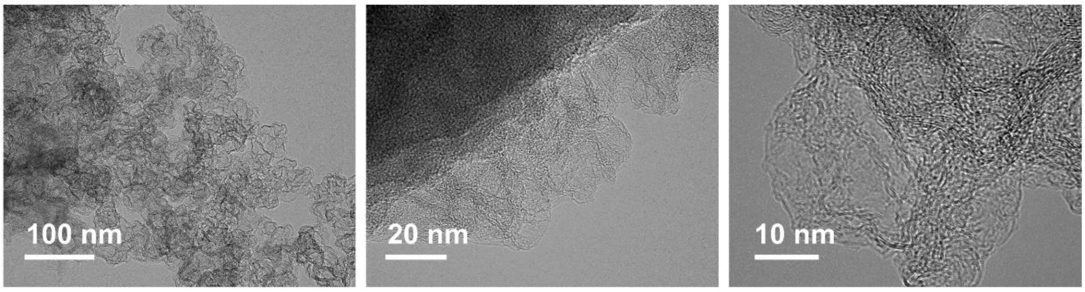

Supplementary Fig. 21. Morphology characterization. The TEM images with different magnifications of CoCOP-CH_3@KB.

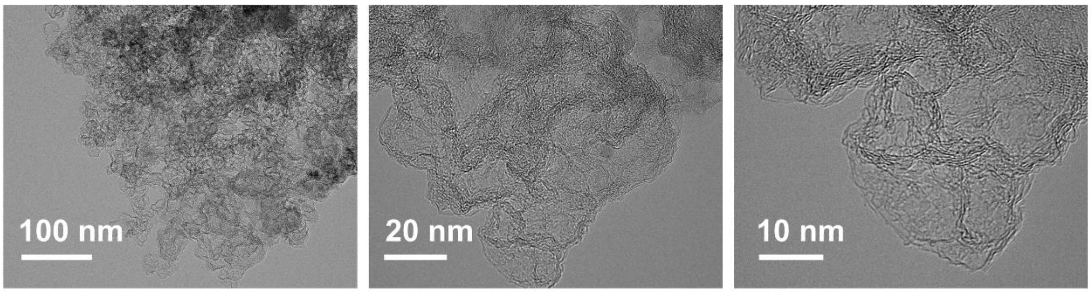

Supplementary Fig. 22. Morphology characterization. The TEM images with different magnifications of CoCOP-H@KB.

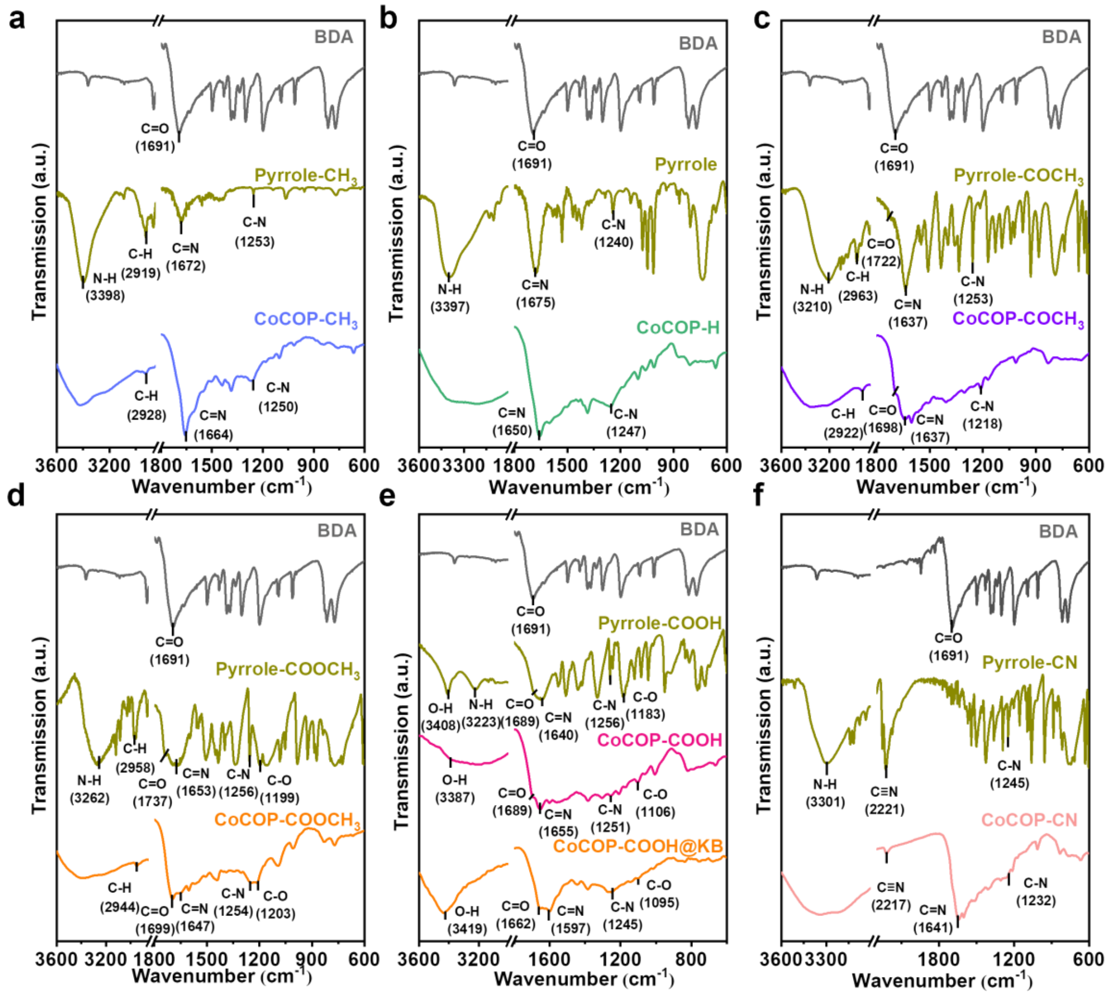

Supplementary Fig. 23. Structural characterization of catalysts. FT-IR spectra of **a** CoCOP-CH_3, **b** CoCOP-H, **c** CoCOP-$COCH_3$, **d** CoCOP-$COOCH_3$, **e** CoCOP-COOH and CoCOP-COOH@KB, **f** CoCOP-CN and their corresponding monomers, including BDA, pyrrole, and substituted-pyrroles.

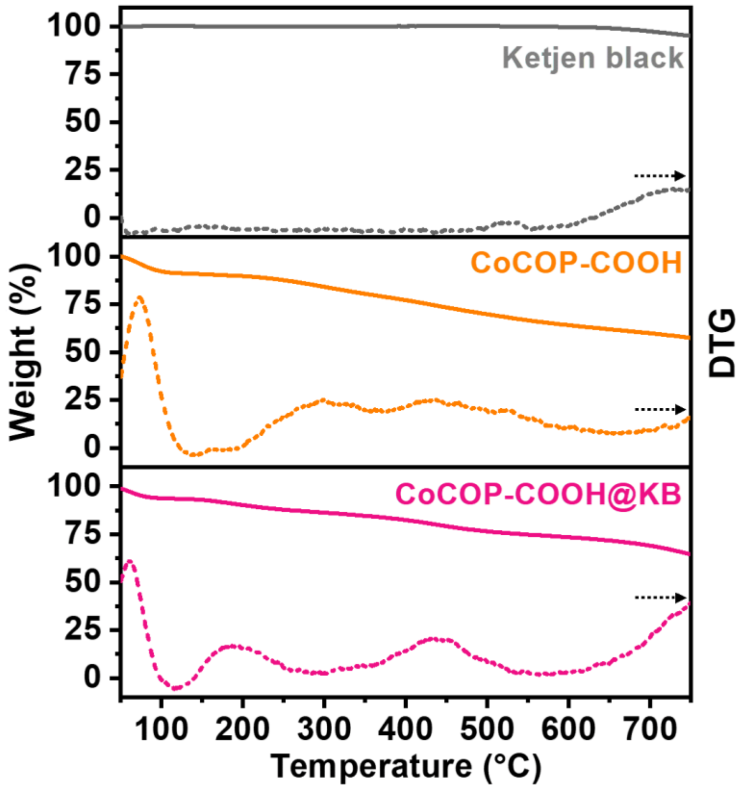

Supplementary Fig. 24. Structural characterization of catalysts. TGA (straight line) and DTG (dotted line) profiles of KB, CoCOP-COOH and CoCOP-COOH@KB.

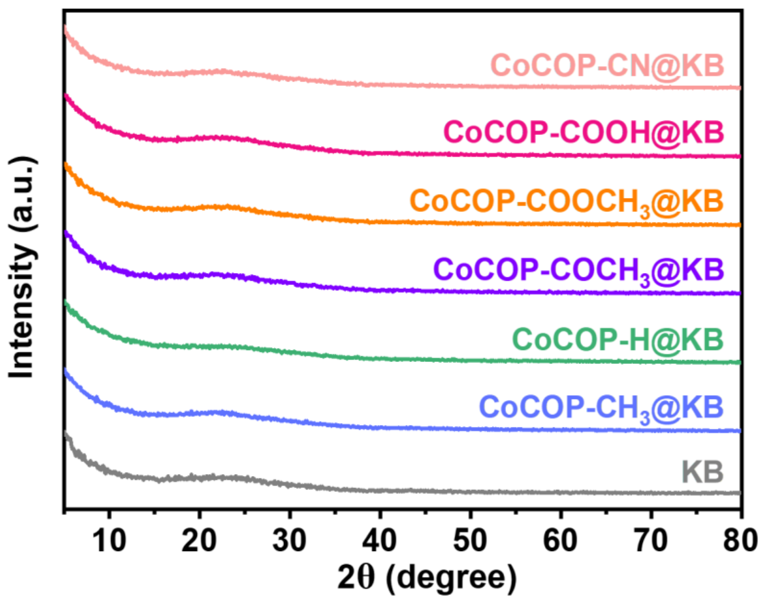

Supplementary Fig. 25. **Structural characterization of catalysts.** XRD patterns of CoCOP-CN@KB, CoCOP-COOH@KB, CoCOP-$COOCH_3$@KB, CoCOP-$COCH_3$@KB, CoCOP-H@KB, CoCOP-CH_3@KB and KB.

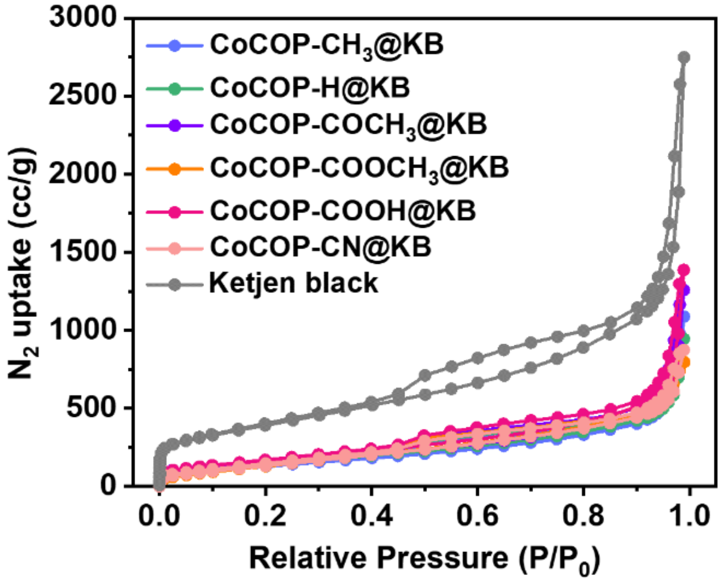

Supplementary Fig. 26. Porous characteristics of catalysts. N_2 adsorption-desorption isotherms of CoCOP-CH_3@KB, CoCOP-H@KB, CoCOP-$COCH_3$@KB, CoCOP-$COOCH_3$@KB, CoCOP-COOH@KB, CoCOP-CN@KB and KB.

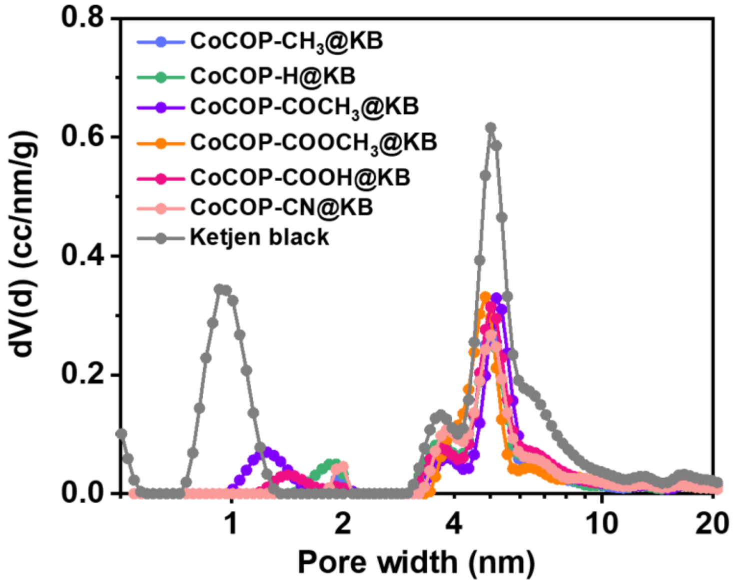

Supplementary Fig. 27. Porous characteristics of catalysts. Pore size distribution profiles of CoCOP-CH_3@KB, CoCOP-H@KB, CoCOP-$COCH_3$@KB, CoCOP-$COOCH_3$@KB, CoCOP-COOH@KB, CoCOP-CN@KB and KB.

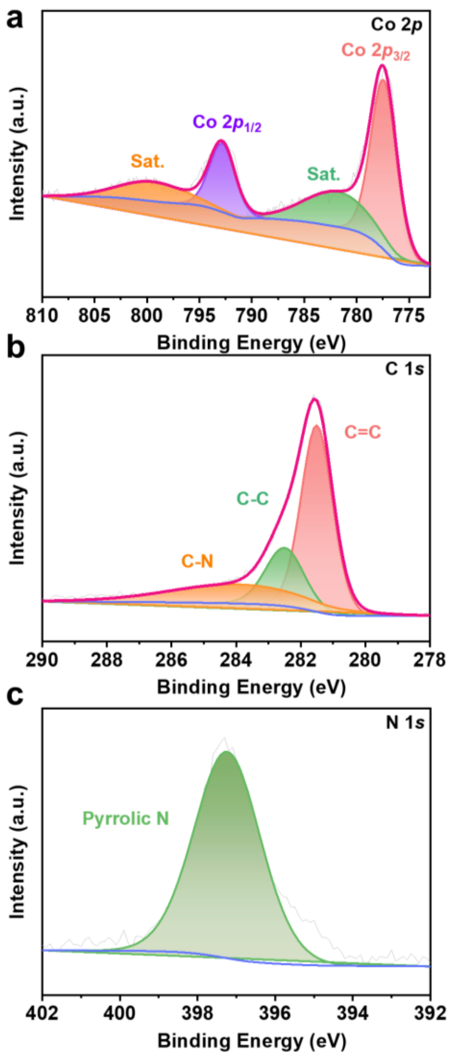

Supplementary Fig. 28. The chemical state characterization. High-resolution **a** Co 2*p*, **b** C 1*s* and **c** N 1*s* XPS spectra of CoCOP-CH_3@KB.

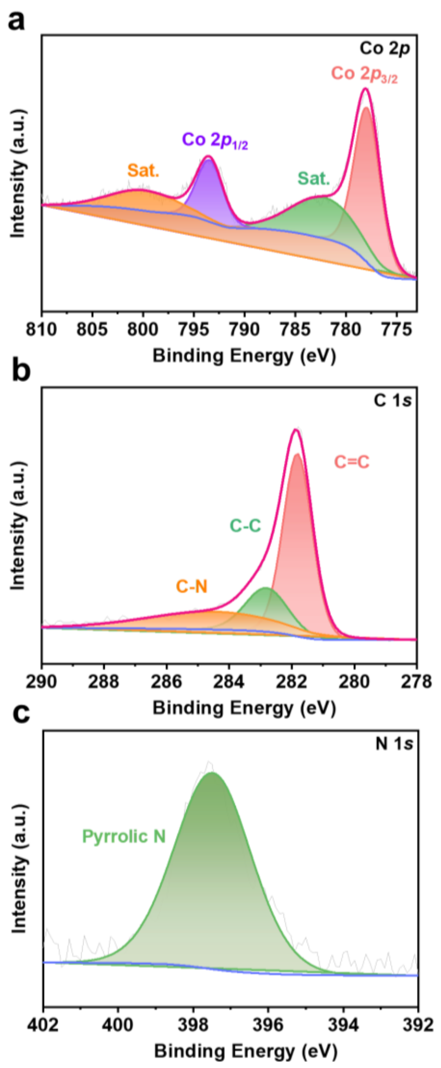

Supplementary Fig. 29. The chemical state characterization. High-resolution **a** Co 2*p*, **b** C 1*s* and **c** N 1*s* XPS spectra of CoCOP-H@KB.

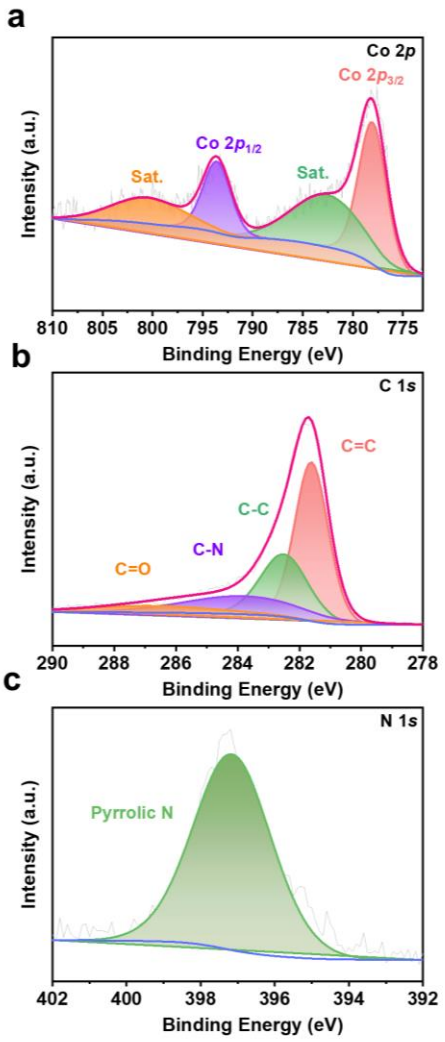

Supplementary Fig. 30. The chemical state characterization. High-resolution **a** Co 2*p*, **b** C 1*s* and **c** N 1*s* XPS spectra of CoCOP-$COCH_3$@KB.

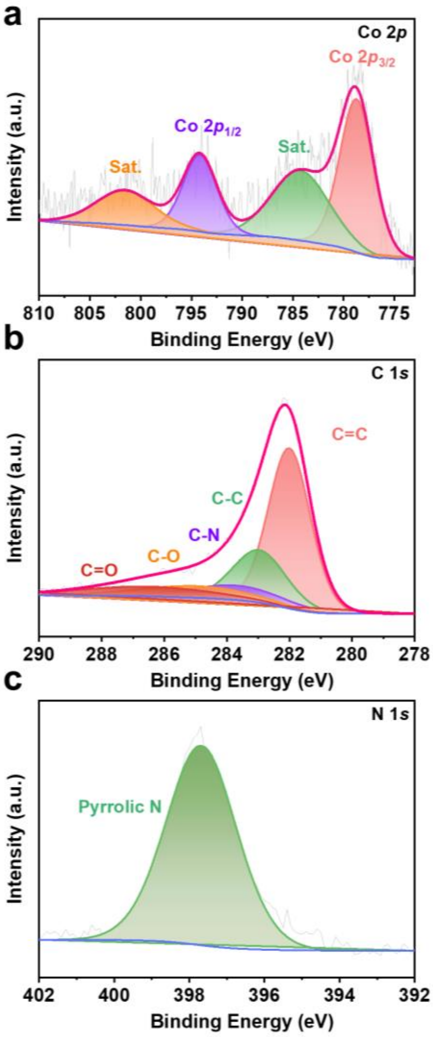

Supplementary Fig. 31. The chemical state characterization. High-resolution **a** Co 2*p*, **b** C 1*s* and **c** N 1*s* XPS spectra of CoCOP-$COOCH_3$@KB.

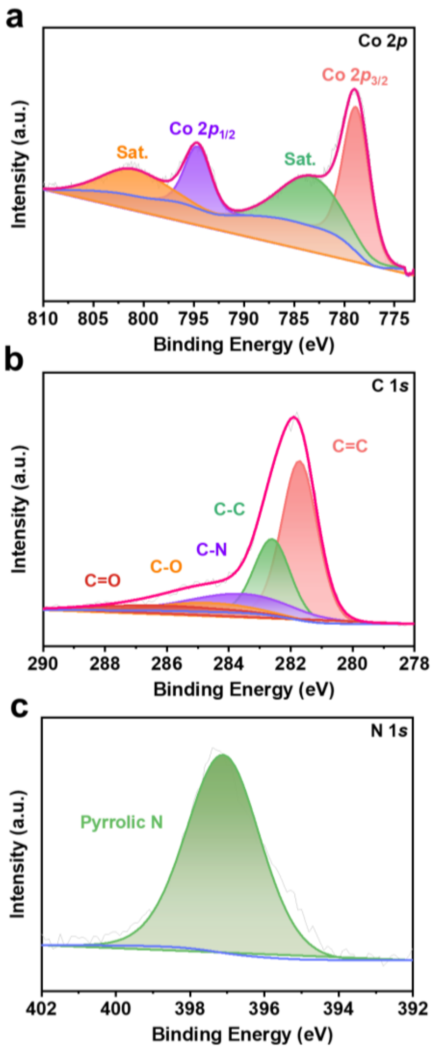

Supplementary Fig. 32. The chemical state characterization. High-resolution **a** Co 2*p*, **b** C 1*s* and **c** N 1*s* XPS spectra of CoCOP-COOH@KB.

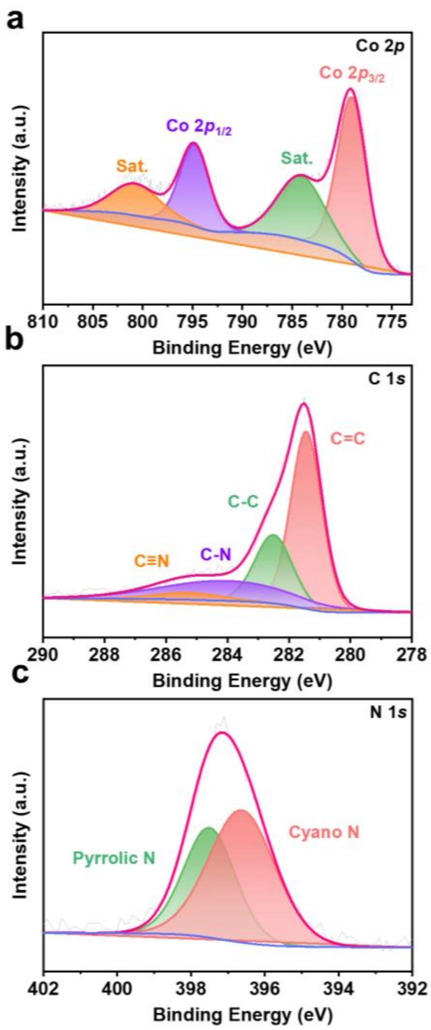

Supplementary Fig. 33. The chemical state characterization. High-resolution **a** Co 2*p*, **b** C 1*s* and **c** N 1*s* XPS spectra of CoCOP-CN@KB.

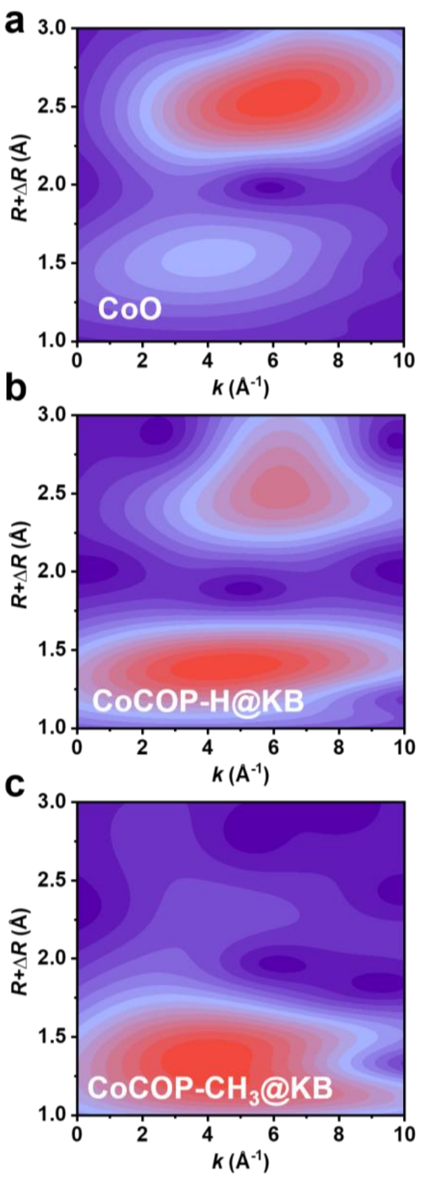

Supplementary Fig. 34. Local environment analysis of Co centers. WT-EXAFS plots of **a** CoO, **b** CoCOP-H@KB and **c** CoCOP-CH_3@KB.

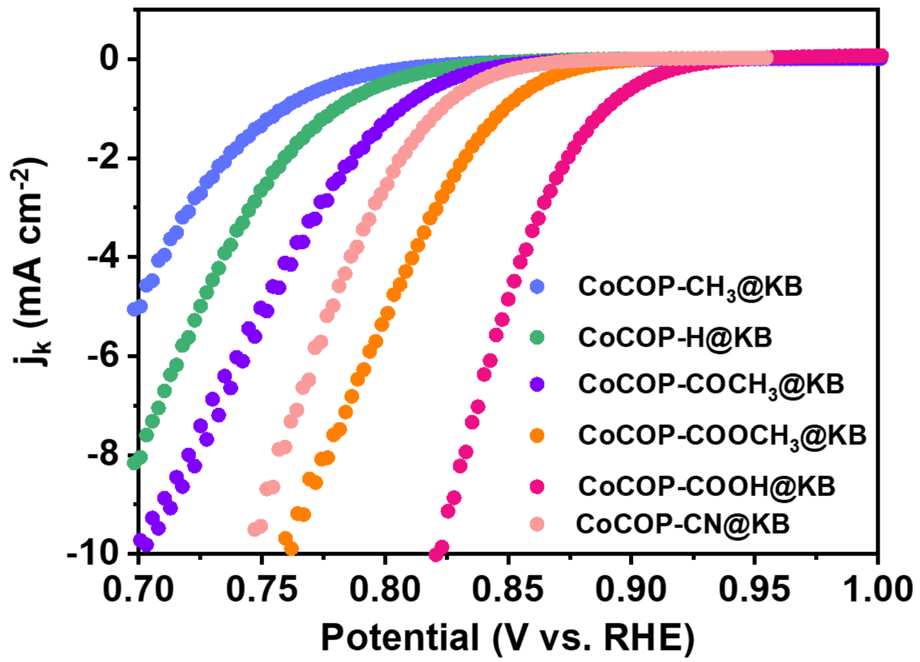

Supplementary Fig. 35. Electrochemical characterization of CoCOP-X@KB catalysts. Kinetic current density curves of CoCOP-CH$_3$@KB, CoCOP-H@KB, CoCOP-COCH$_3$@KB, CoCOP-COOCH$_3$@KB, CoCOP-COOH@KB and CoCOP-CN@KB.

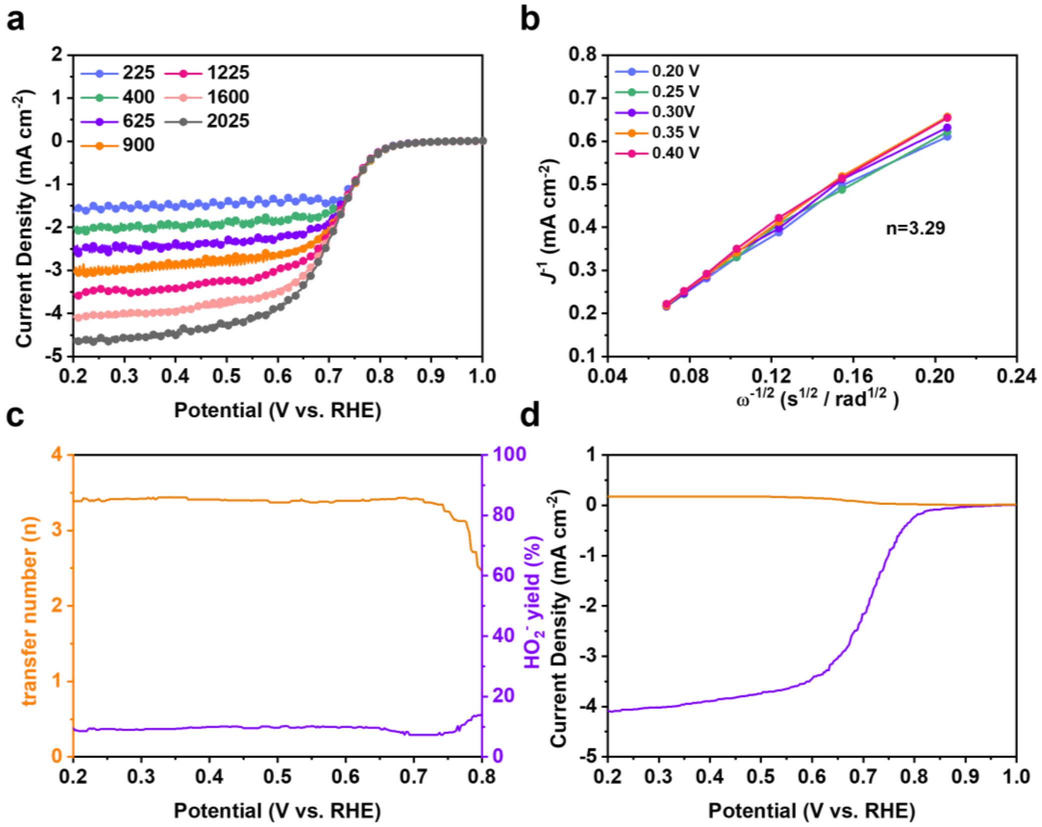

Supplementary Fig. 36. Characterization of catalytic activity and selectivity. a RDE LSV curves at different rotation speeds, **b** the corresponding K-L plots at different potentials, **c** the HO_2^- yield and electron transfer number, **d** RRDE LSV curve of CoCOP-CH_3@KB.

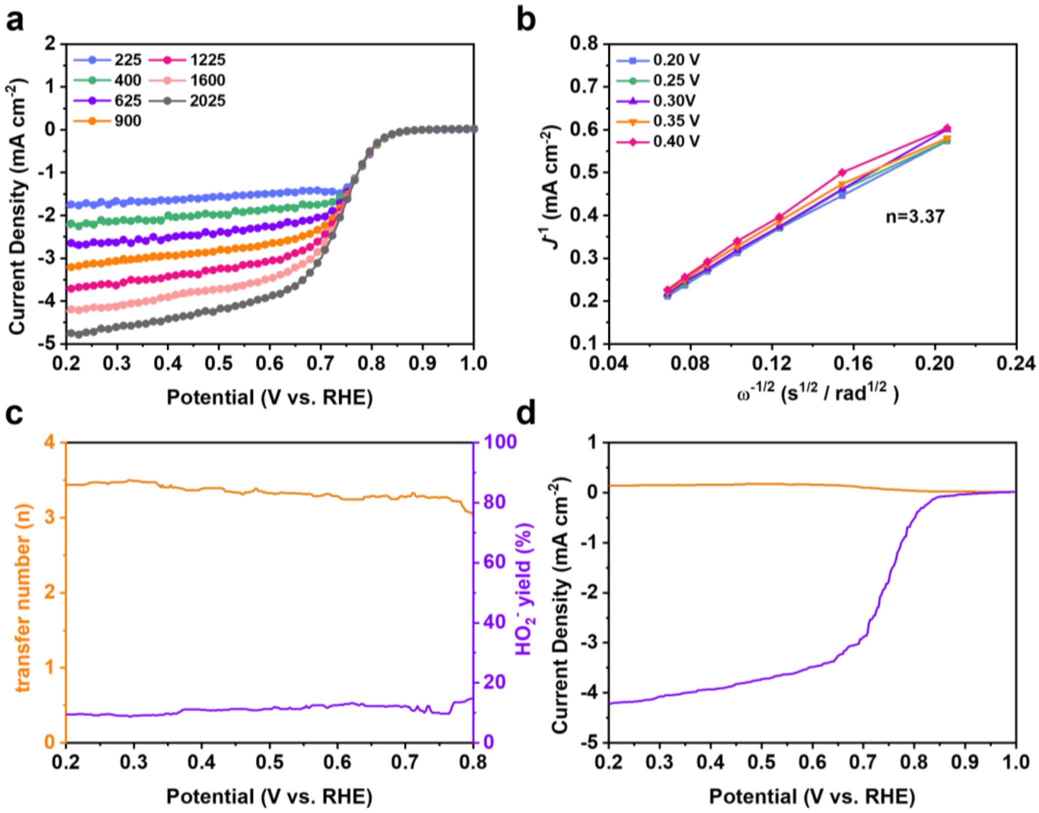

Supplementary Fig. 37. Characterization of catalytic activity and selectivity. a RDE LSV curves at different rotation speeds, **b** the corresponding K-L plots at different potentials, **c** the HO_2^- yield and electron transfer number, **d** RRDE LSV curve of CoCOP-H@KB.

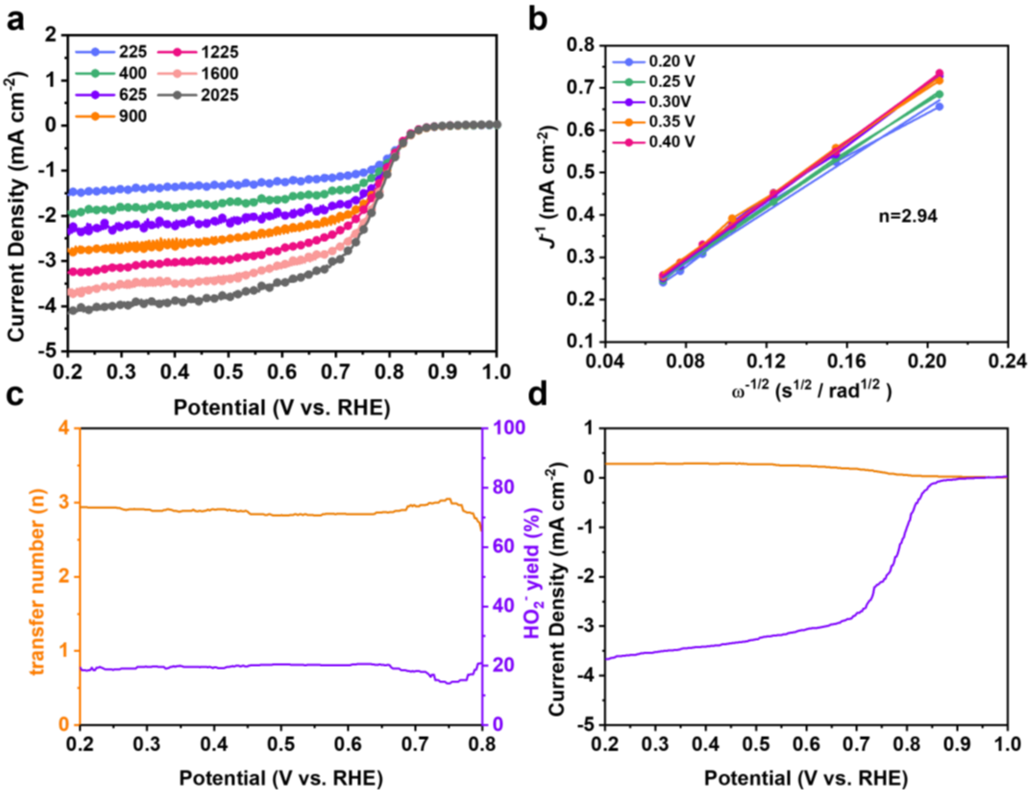

Supplementary Fig. 38. Characterization of catalytic activity and selectivity. a RDE LSV curves at different rotation speeds, **b** the corresponding K-L plots at different potentials, **c** the HO_2^- yield and electron transfer number, **d** RRDE LSV curve of CoCOP-$COCH_3$@KB.

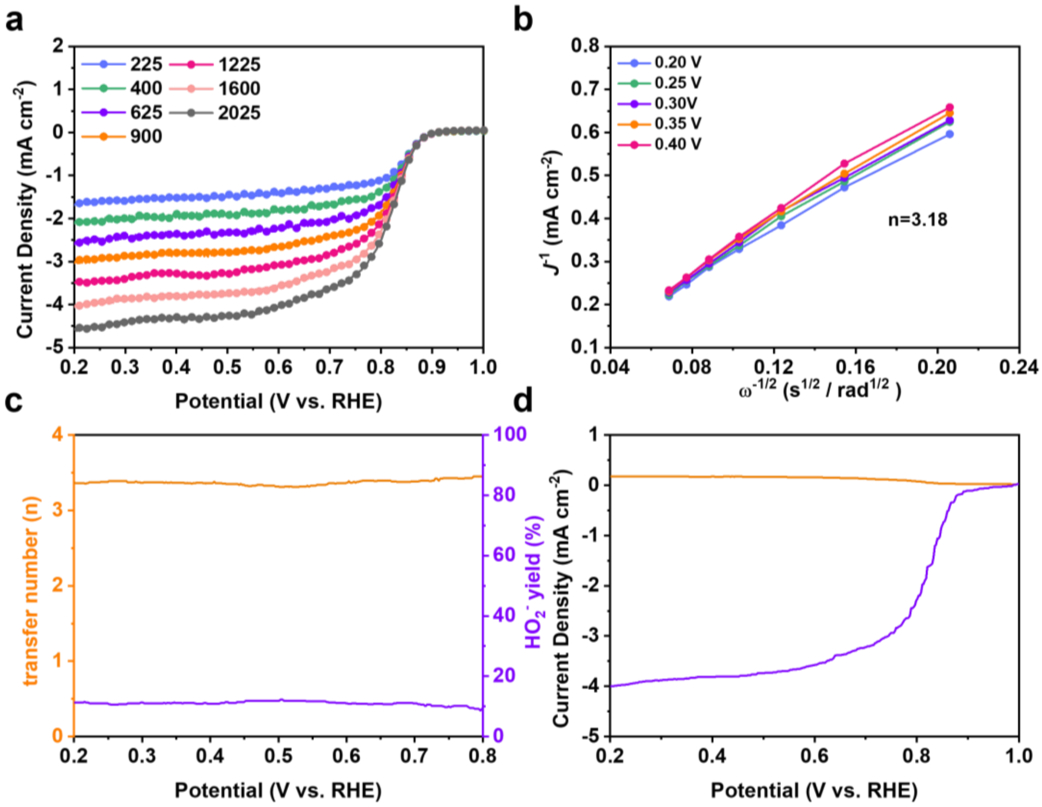

Supplementary Fig. 39. Characterization of catalytic activity and selectivity. a RDE LSV curves at different rotation speeds, **b** the corresponding K-L plots at different potentials, **c** the HO_2^- yield and electron transfer number, **d** RRDE LSV curve of CoCOP-COOCH$_3$@KB.

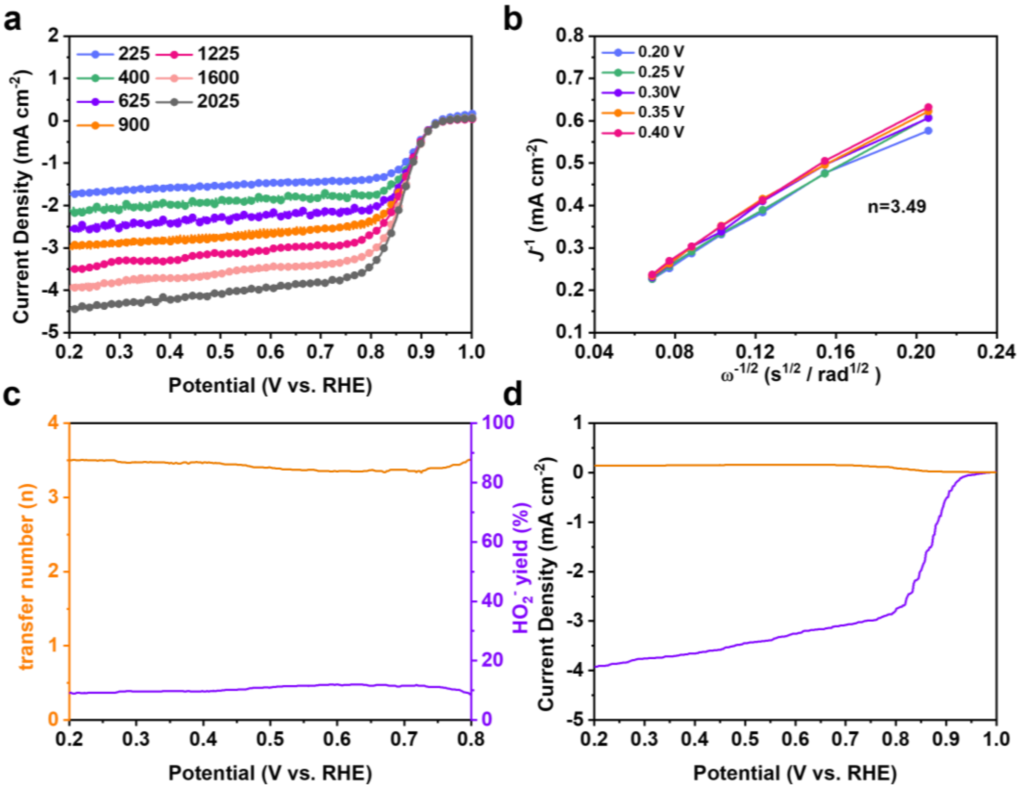

Supplementary Fig. 40. Characterization of catalytic activity and selectivity. a RDE LSV curves at different rotation speeds, **b** the corresponding K-L plots at different potentials, **c** the HO_2^- yield and electron transfer number, **d** RRDE LSV curve of CoCOP-COOH@KB.

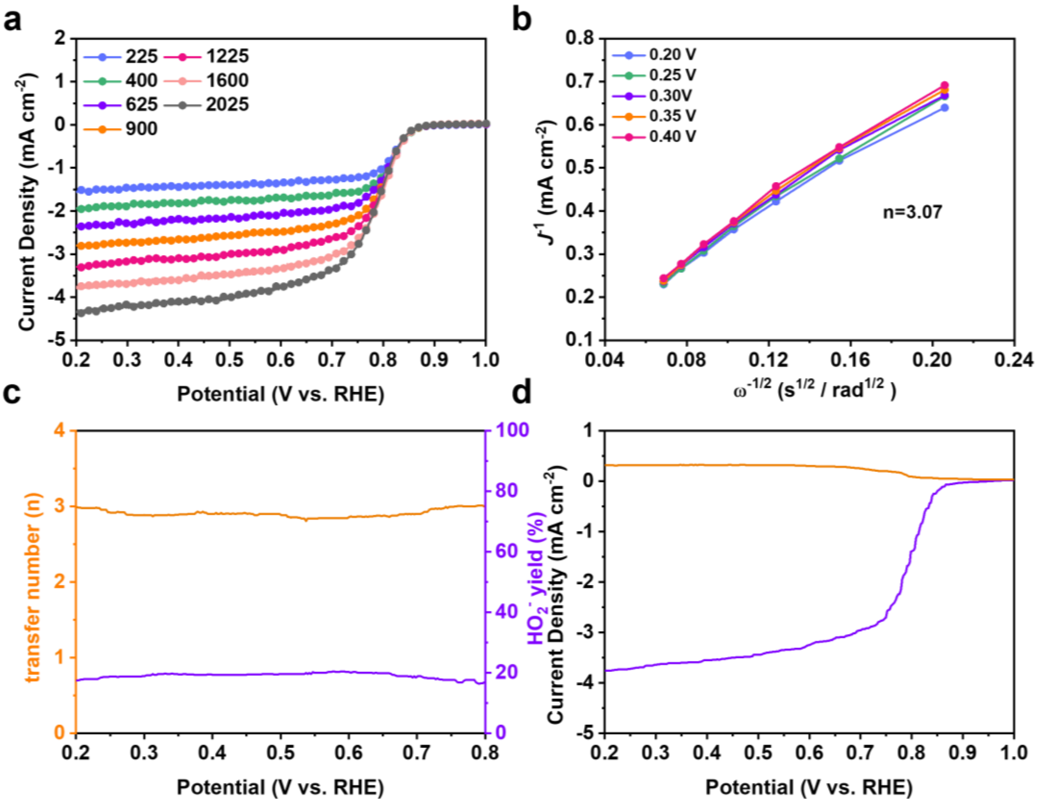

Supplementary Fig. 41. Characterization of catalytic activity and selectivity. a RDE LSV curves at different rotation speeds, **b** the corresponding K-L plots at different potentials, **c** the HO_2^- yield and electron transfer number, **d** RRDE LSV curve of CoCOP-CN@KB.

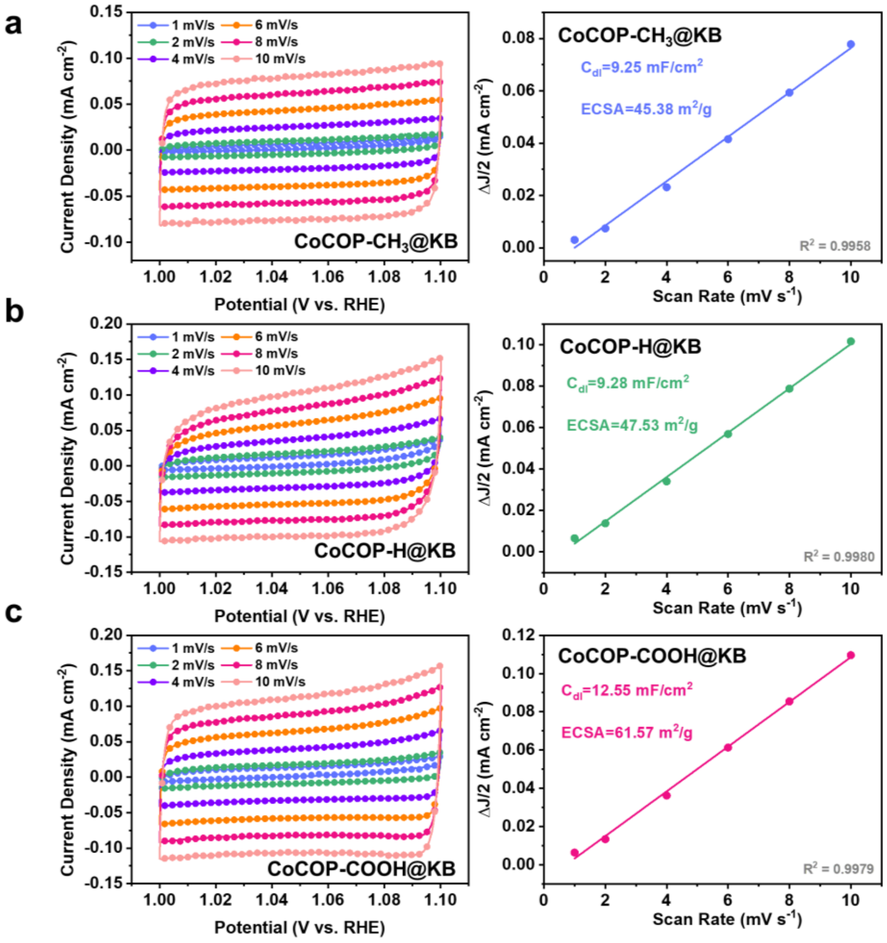

Supplementary Fig. 42. ECSA analysis. CV curves in the region of 1.0-1.1 V (vs. RHE) at various scan rates (left), the corresponding double-layer capacitance (C_{dl}) and ECSA (right) of **a** CoCOP-CH_3@KB, **b** CoCOP-H@KB and **c** CoCOP-COOH@KB at room temperature (~25 °C).

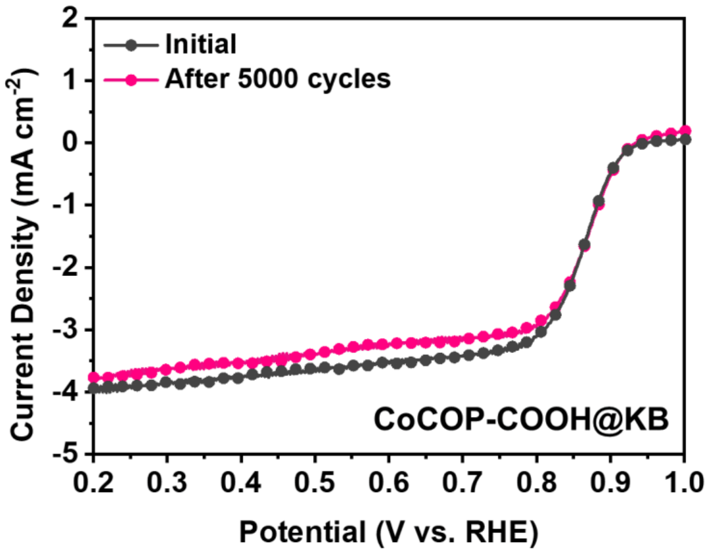

Supplementary Fig. 43. Long-term stability test. The LSV polarization curves of CoCOP-COOH@KB catalyst before and after 5000 cycles.

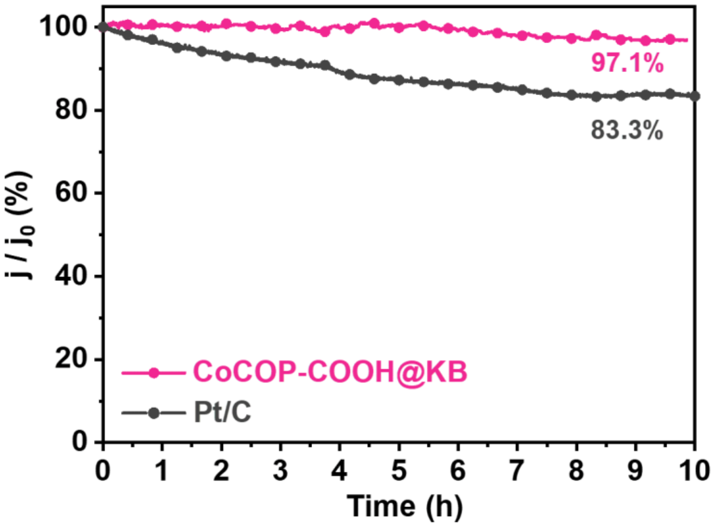

Supplementary Fig. 44. Long-term stability test. Chronoamperometric responses of CoCOP-COOH@KB and Pt/C at 0.4 V (vs. RHE) for 10 h.

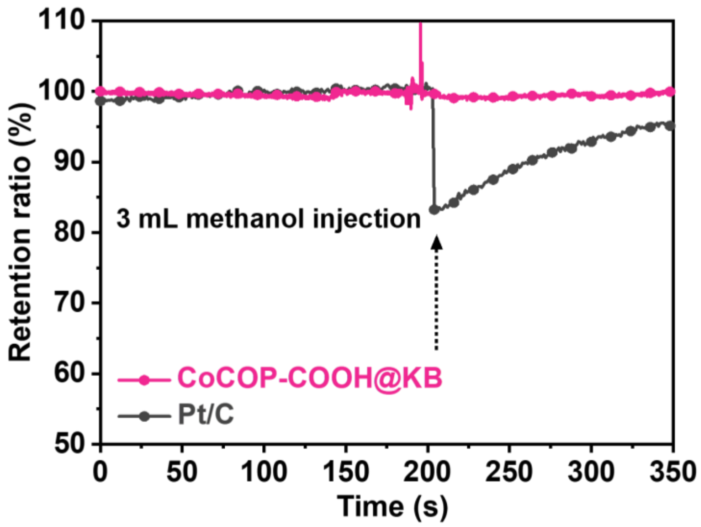

Supplementary Fig. 45. Methanol crossover test. Chronoamperometric responses of CoCOP-COOH@KB and Pt/C at 0.4 V with the injection of 3 mL methanol.

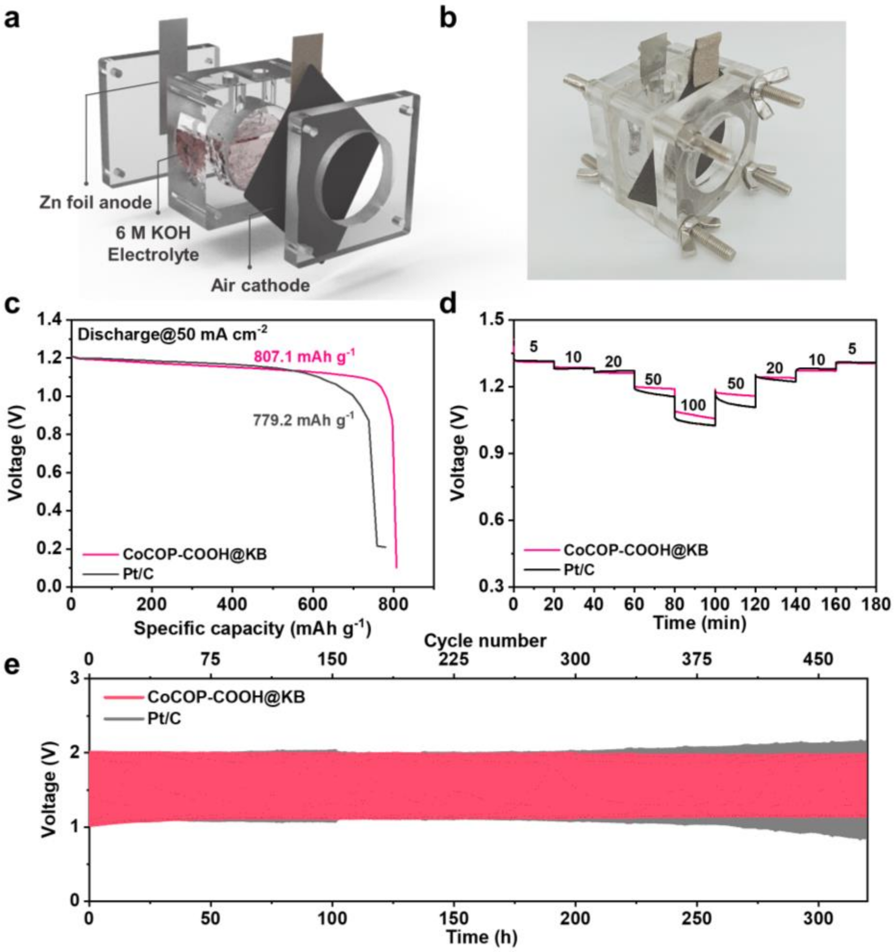

Supplementary Fig. 46. ZAB device performance. a Schematic configuration of ZAB device. **b** Photographic image of assembled ZAB device. **c** Galvanostatic discharge curves at the current density of 50 mA cm^{-2} in the first discharge cycle, **d** galvanostatic discharge process at various current densities. **e** Galvanostatic cycling stability at 25 mA cm^{-2} of ZABs using CoCOP-COOH@KB and Pt/C as cathode catalysts at room temperature (~25 ℃).

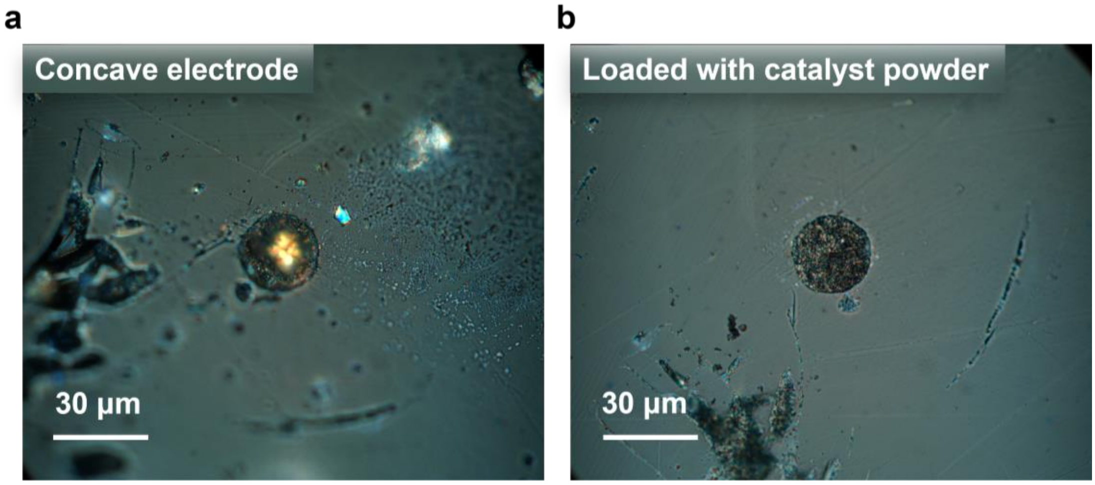

Supplementary Fig. 47. The catalyst loading on Au UME detected by optical microscope. Optical microscope images of **a** clean concave electrode and **b** concave electrode loaded with catalyst powder from vertical view.

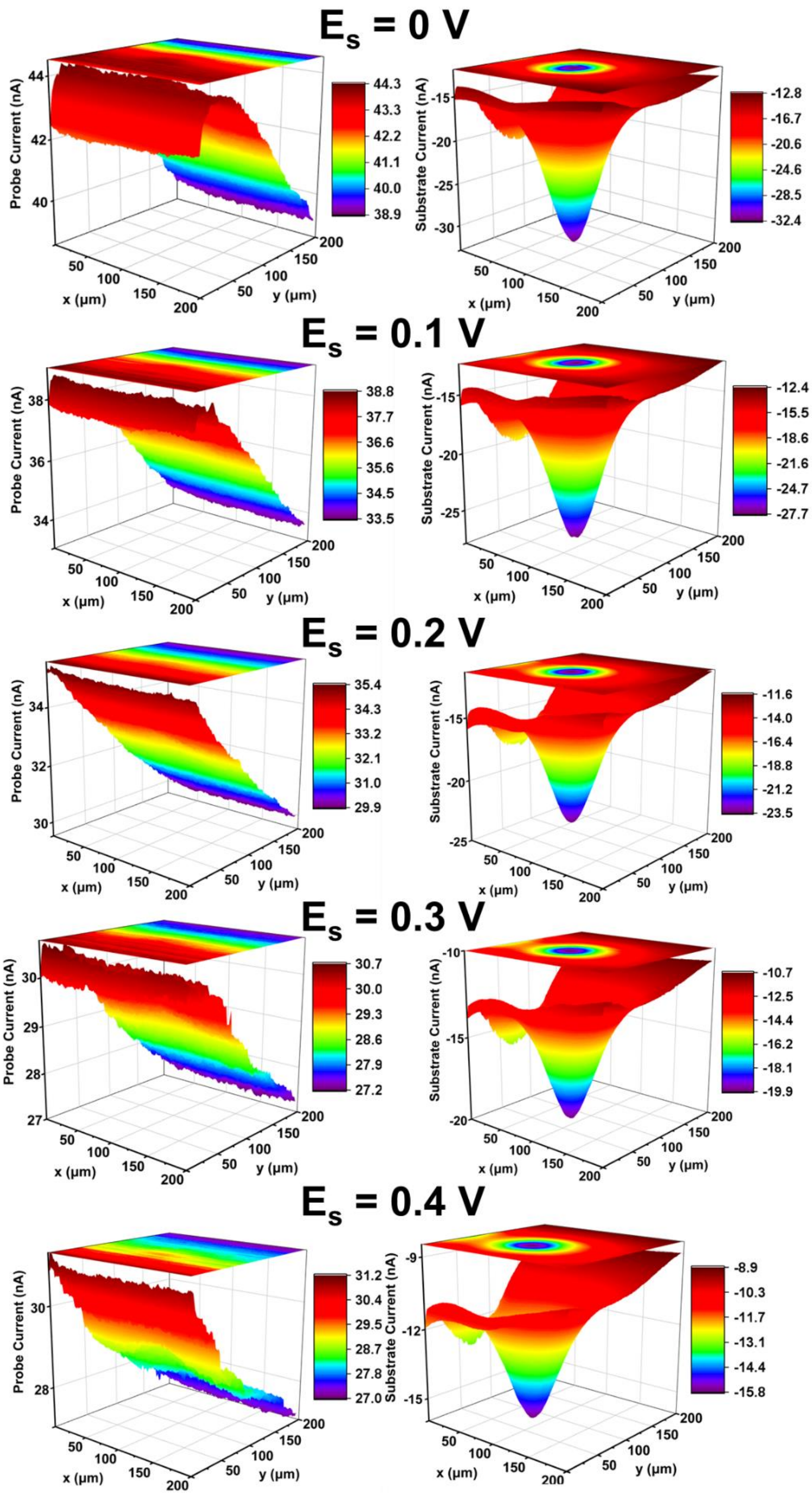

Supplementary Fig. 48. The local catalytic reactivity obtained by SECM technology. TG/SC mode SECM images of CoCOP-CH_3@KB at substrate potential of 0.0 – 0.9 V (vs. RHE). The probe is set at a certain potential of 1.8146 V (vs. RHE).

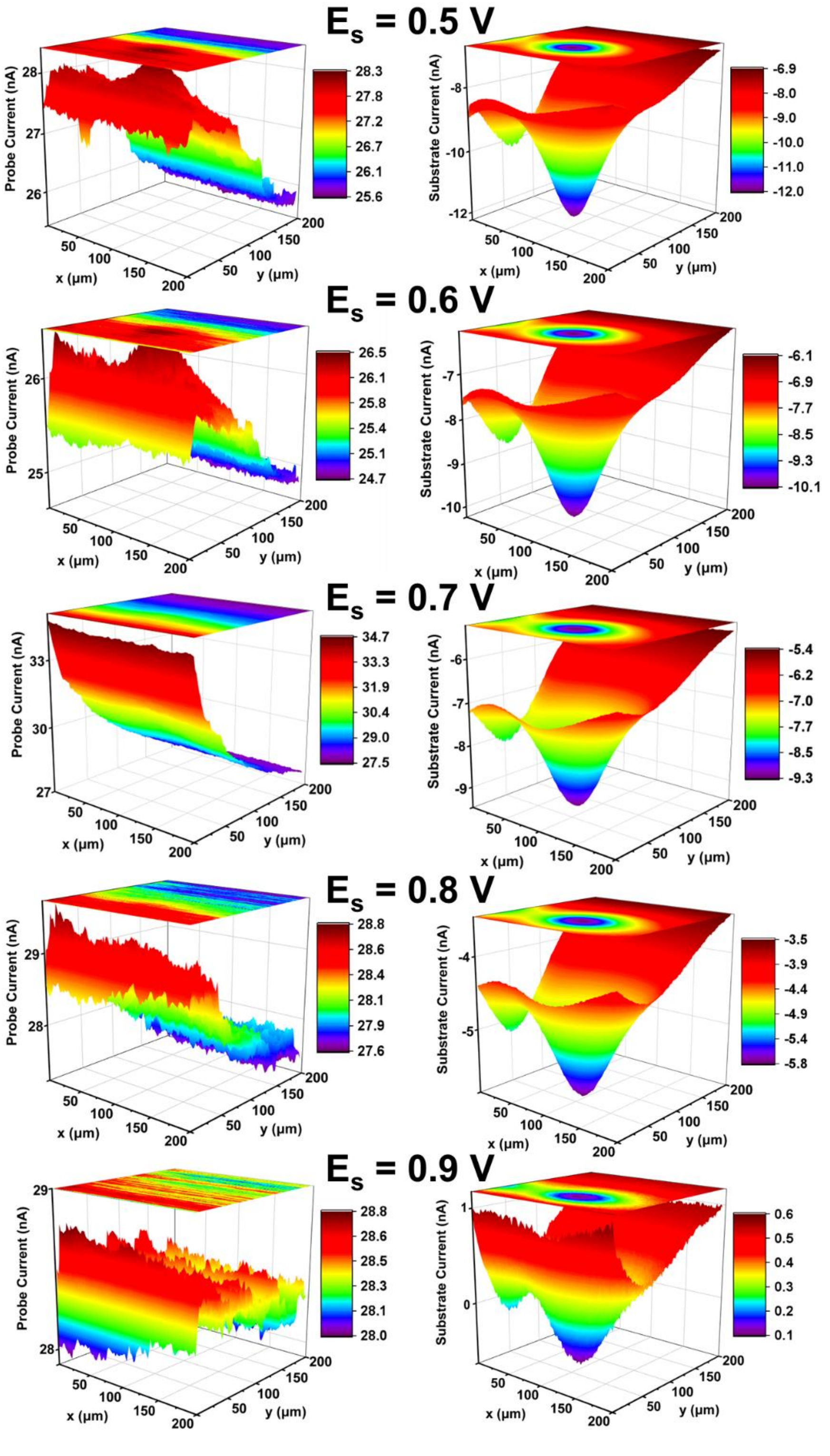

Supplementary Fig. 48. Continued.

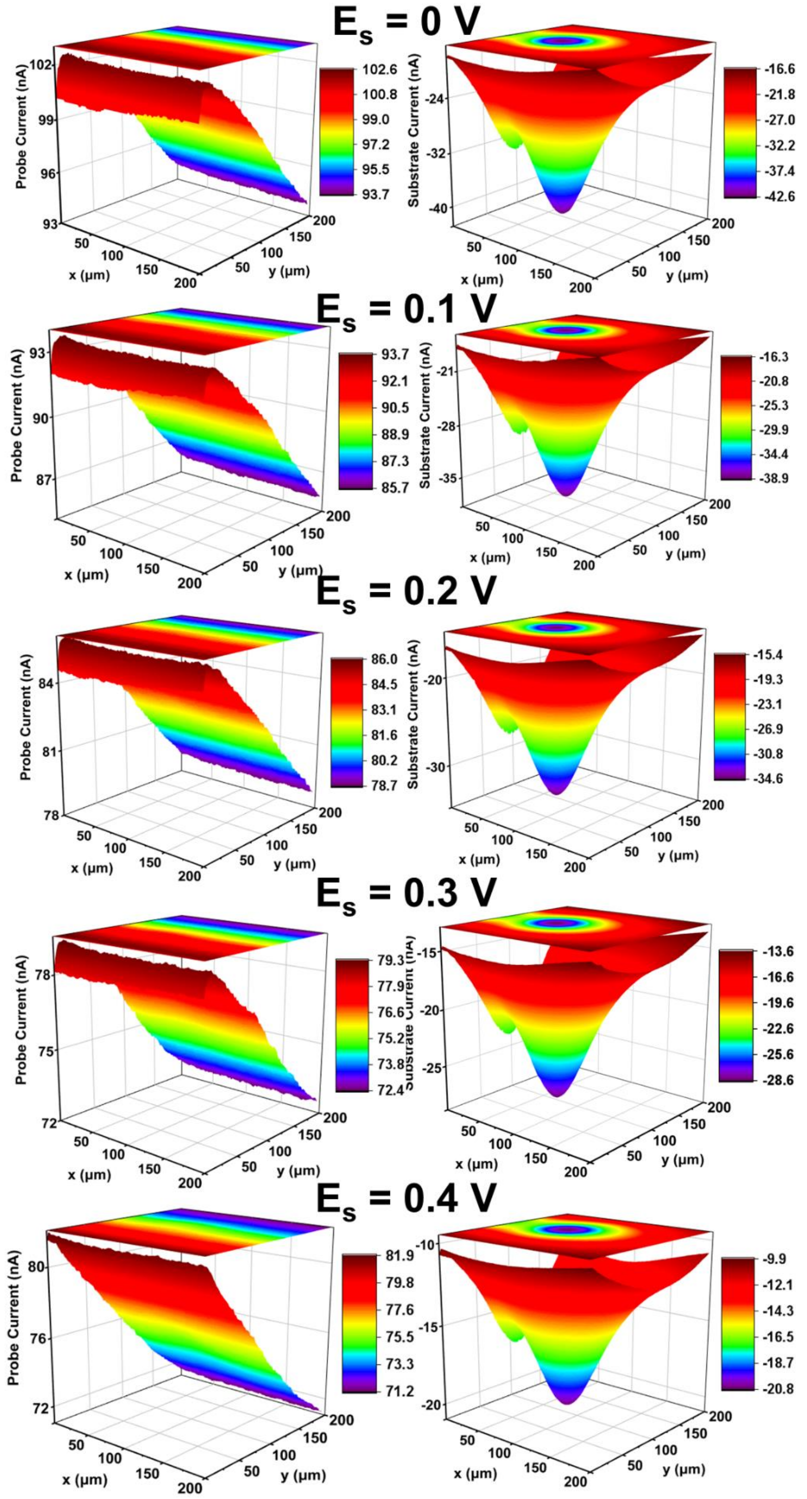

Supplementary Fig. 49. The local catalytic reactivity obtained by SECM technology. TG/SC mode SECM images of CoCOP-H@KB at substrate potential of 0.0 – 0.9 V (vs. RHE). The probe is set at a certain potential of 1.8146 V (vs. RHE).

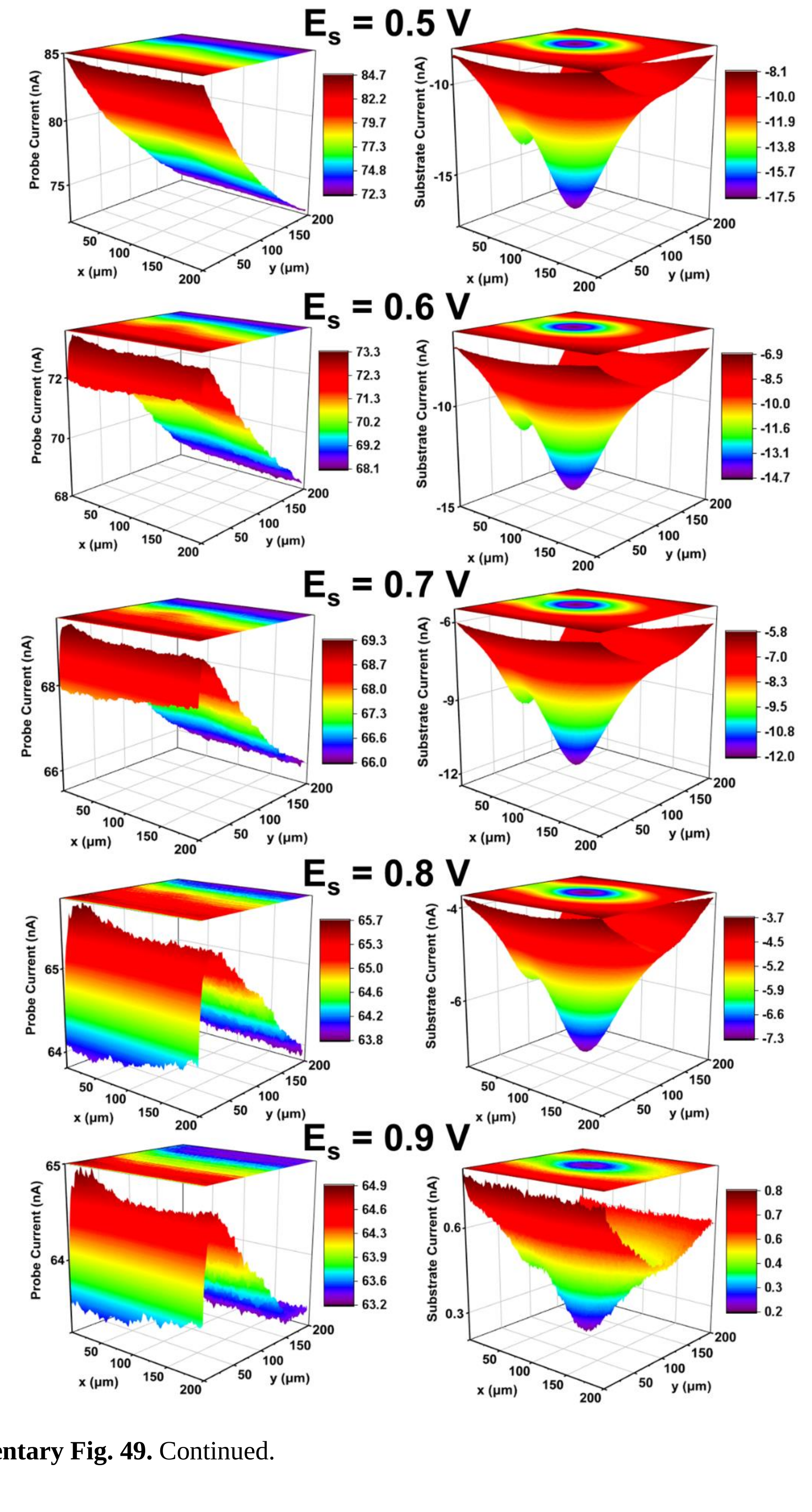

Supplementary Fig. 49. Continued.

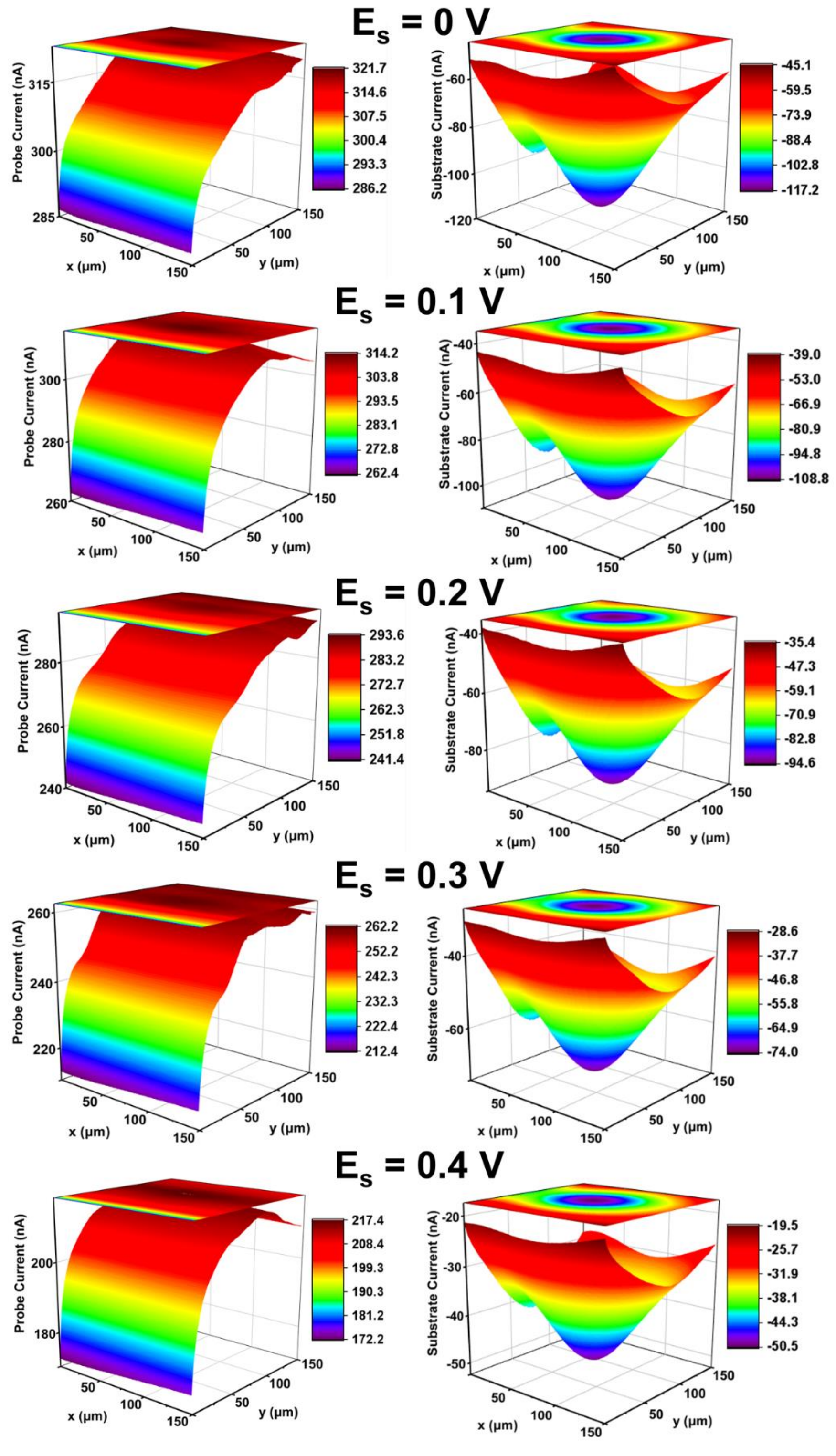

Supplementary Fig. 50. The local catalytic reactivity obtained by SECM technology. TG/SC mode SECM images of CoCOP-COOH@KB at substrate potential of 0.0 – 0.9 V (vs. RHE). The probe is set at a certain potential of 1.8146 V (vs. RHE).

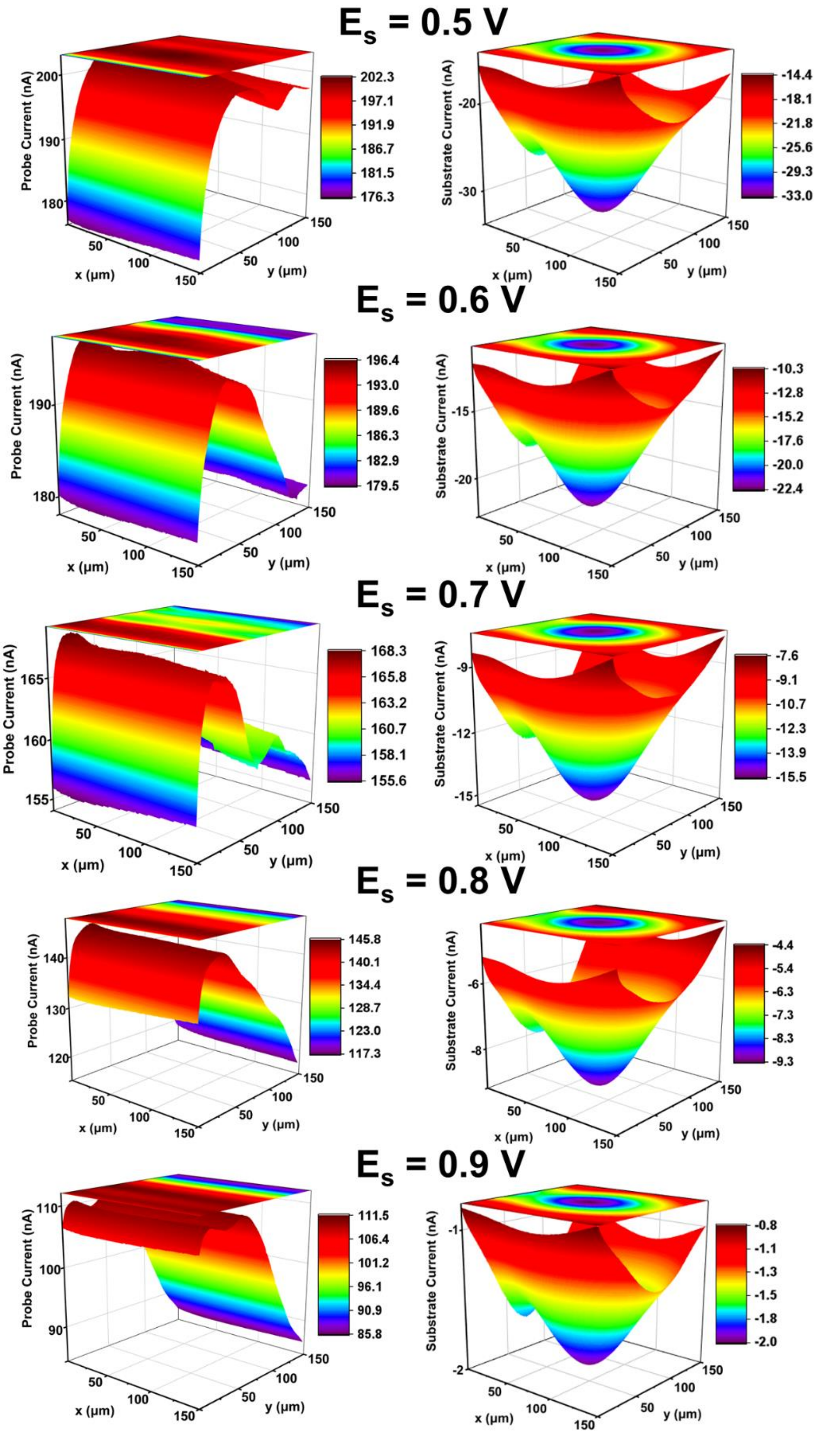

Supplementary Fig. 50. Continued.

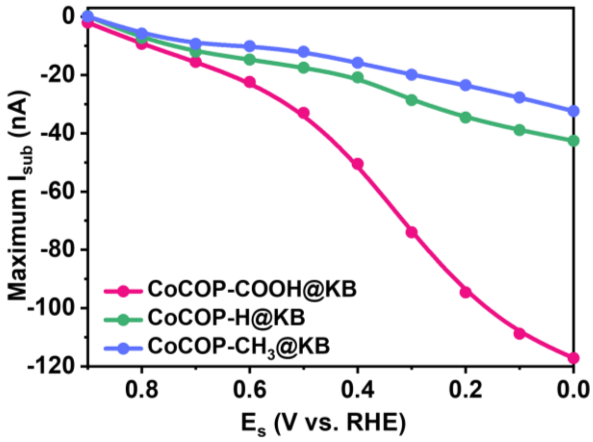

Supplementary Fig. 51. The local catalytic reactivity obtained by SECM technology. Plots of maximum substrate current as functions of substrate potential for SECM results.

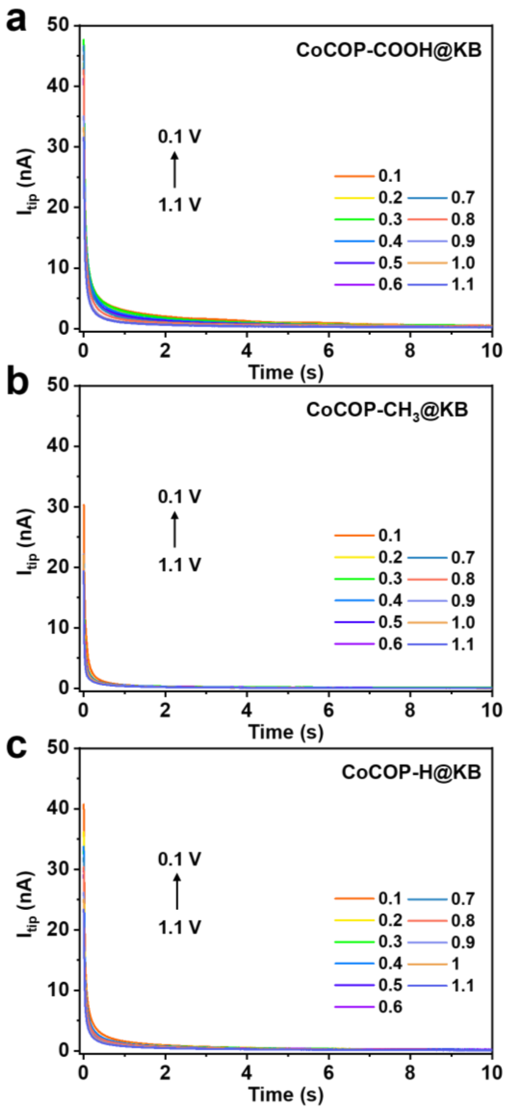

Supplementary Fig. 52. The current response caused by the $FcMeOH^+$/FcMeOH transition obtained by SI-SECM technology. Chronoamperometry curves recorded on the tip in SI-SECM measurement of **a** CoCOP-COOH@KB, **b** CoCOP-CH_3@KB and **c** CoCOP-H@KB.

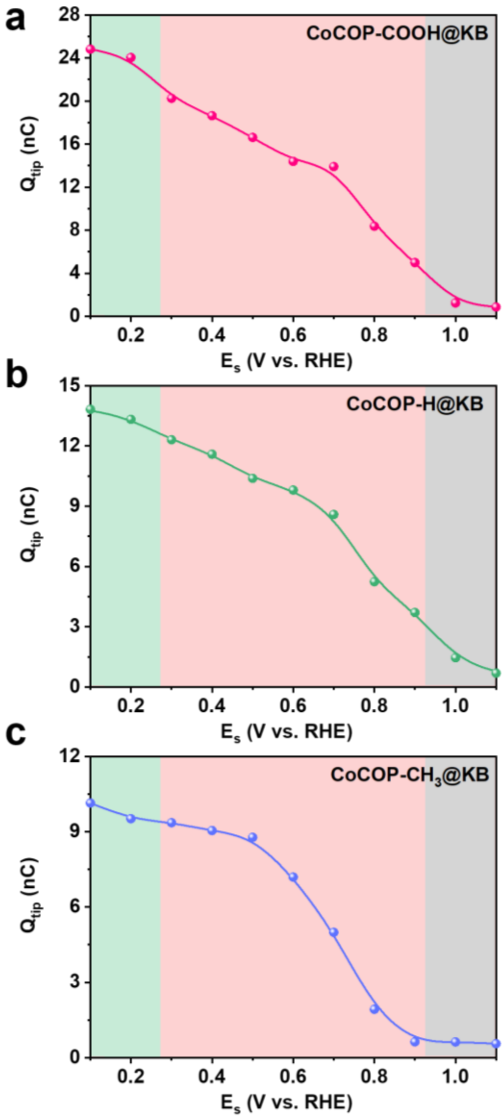

Supplementary Fig. 53. The integrated tip-collected electric charge quantity obtained by SI-SECM technology. Integrated charge with SI-SECM of **a** CoCOP-COOH@KB, **b** CoCOP-H@KB and **c** CoCOP-CH_3@KB.

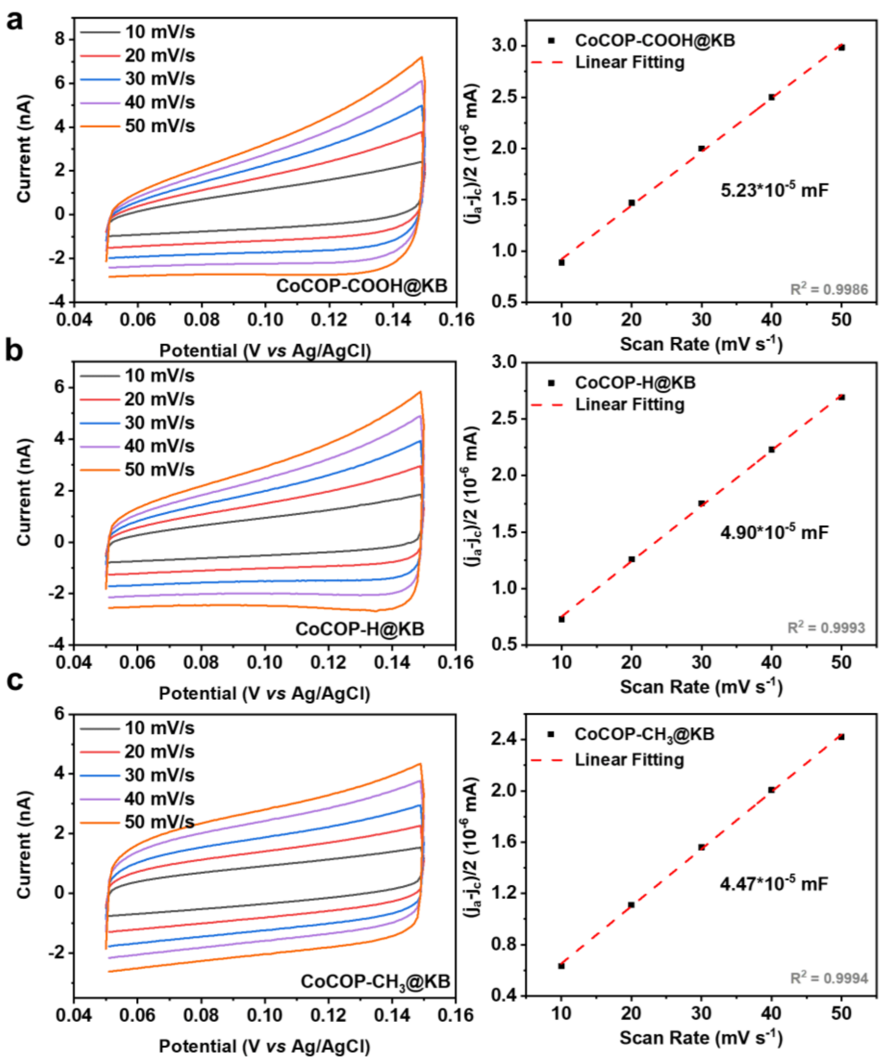

Supplementary Fig. 54. ECSA analysis on UME. CV curves at different scan rates (left) and corresponding fitting results of C_{dl} (right) of **a** CoCOP-COOH@KB, **b** CoCOP-H@KB and **c** CoCOP-CH_3@KB at room temperature (~25 °C).

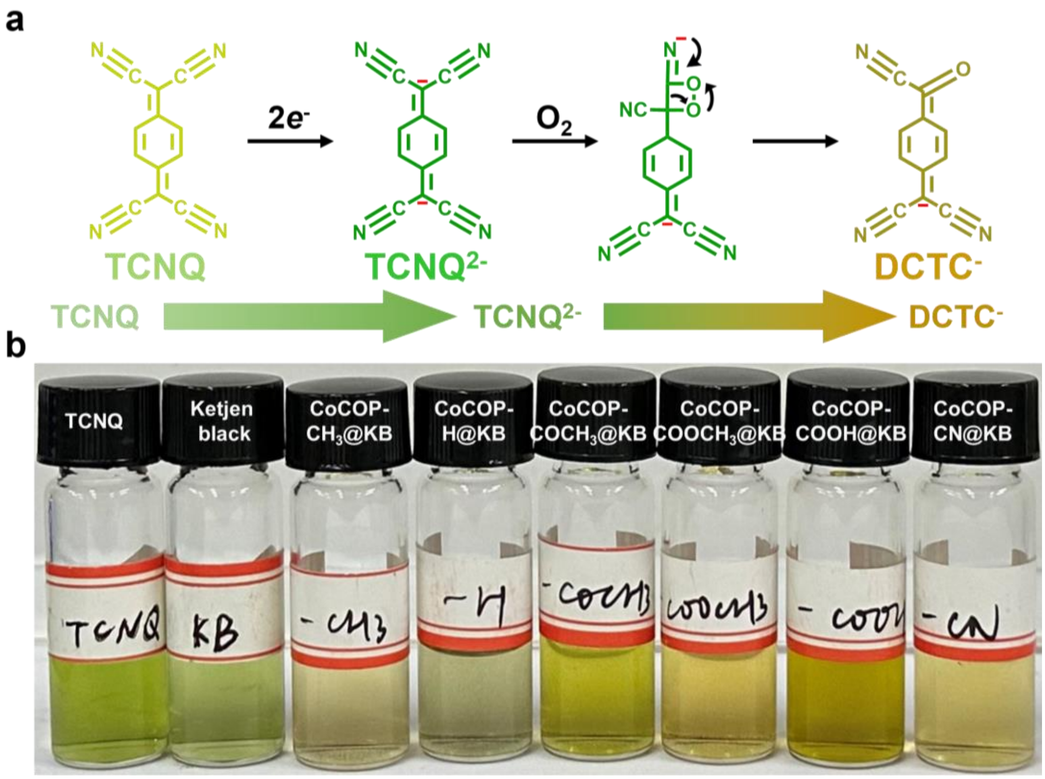

Supplementary Fig. 55. Interfacial charge transfer mechanism analysis. a Electron transfer reaction of TCNQ. **b** Optical image of TCNQ solutions in acetonitrile, and with KB and CoCOP-X@KB nanocomposite analogs.

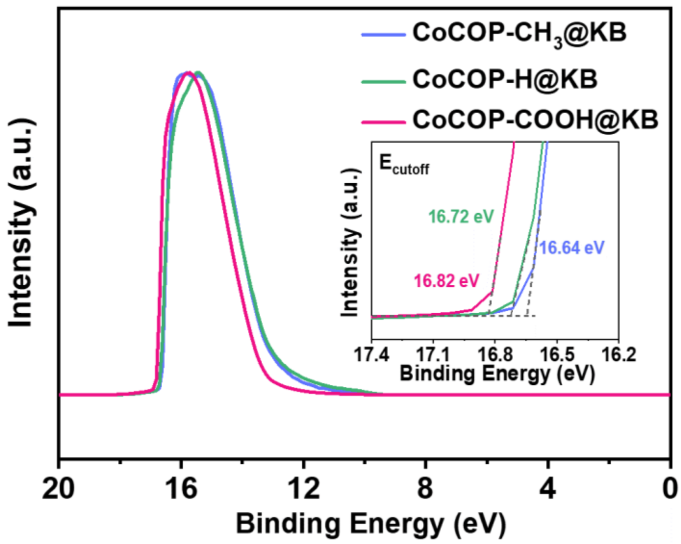

Supplementary Fig. 56. Electronic structure and work function analysis. The ultraviolet photoemission spectra of CoCOP-COOH@KB, CoCOP-H@KB and CoCOP-CH_3@KB; inset shows narrower region of the same spectra. The work function (ϕ) can be obtained according to $\phi = h\nu - E_{cutoff}$.

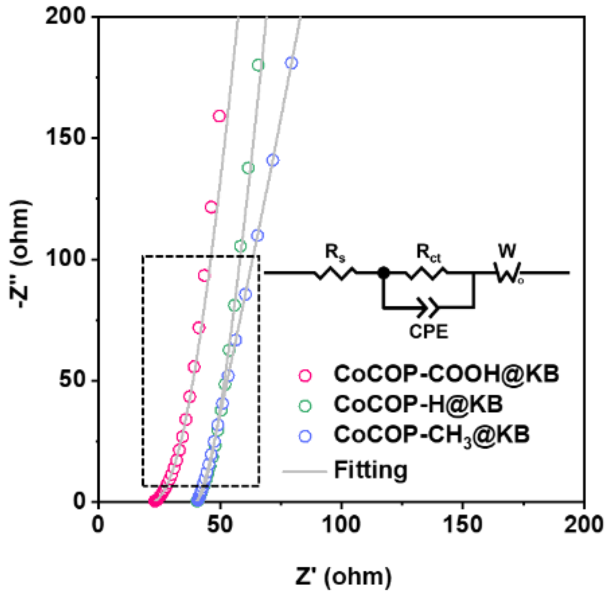

Supplementary Fig. 57. Electron transfer analysis between catalysts and electrolyte. EIS Nyquist comparison plots of CoCOP-COOH@KB, CoCOP-H@KB and CoCOP-CH_3@KB at room temperature (~25 °C) with the marked zone of Warburg resistance. The raw impedance data are represented by symbols and the fitted data are represented by lines. Inset: the equivalent circuit model. R_s, solution resistance. R_{ct}, charge transfer resistance. W, Warburg resistance.

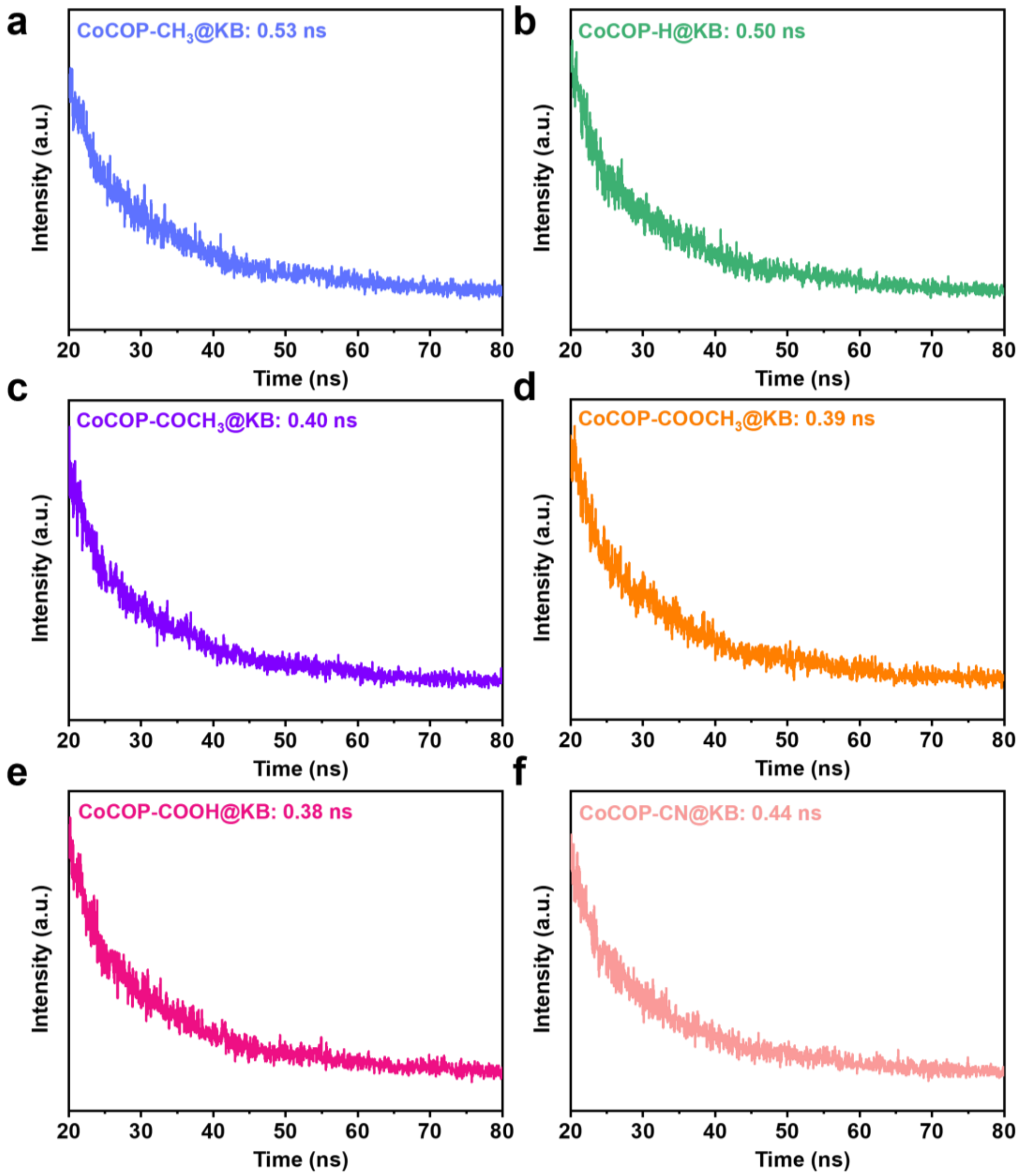

Supplementary Fig. 58. Electron transfer analysis. Fluorescent quantum lifetimes of **a** CoCOP-CH_3@KB, **b** CoCOP-H@KB, **c** CoCOP-$COCH_3$@KB, **d** CoCOP-$COOCH_3$@KB, **e** CoCOP-COOH@KB and **f** CoCOP-CN@KB.

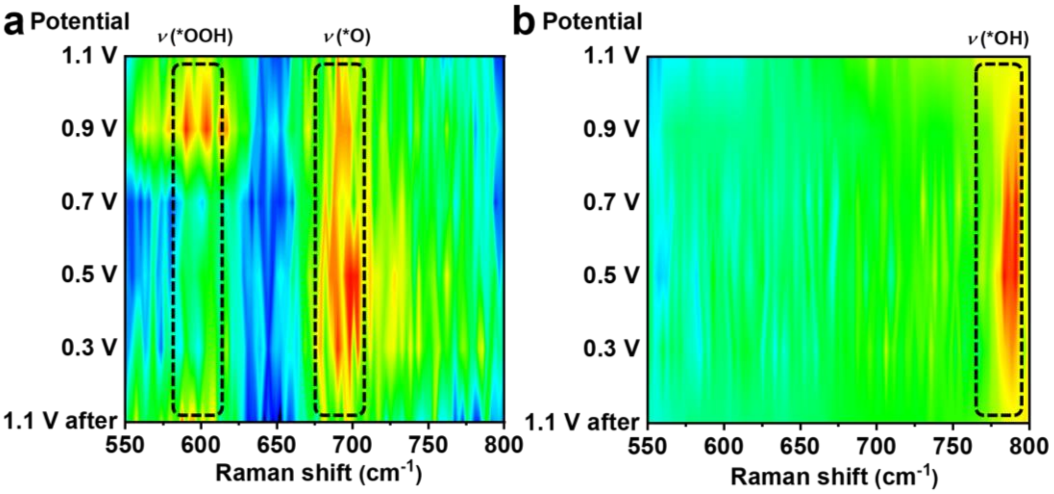

Supplementary Fig. 59. Probing the site transformation by in situ Raman technology. Contour-type in situ Raman spectra of **a** CoCOP-H@KB electrode and **b** CoCOP-CH_3@KB electrode at room temperature (~25 °C).

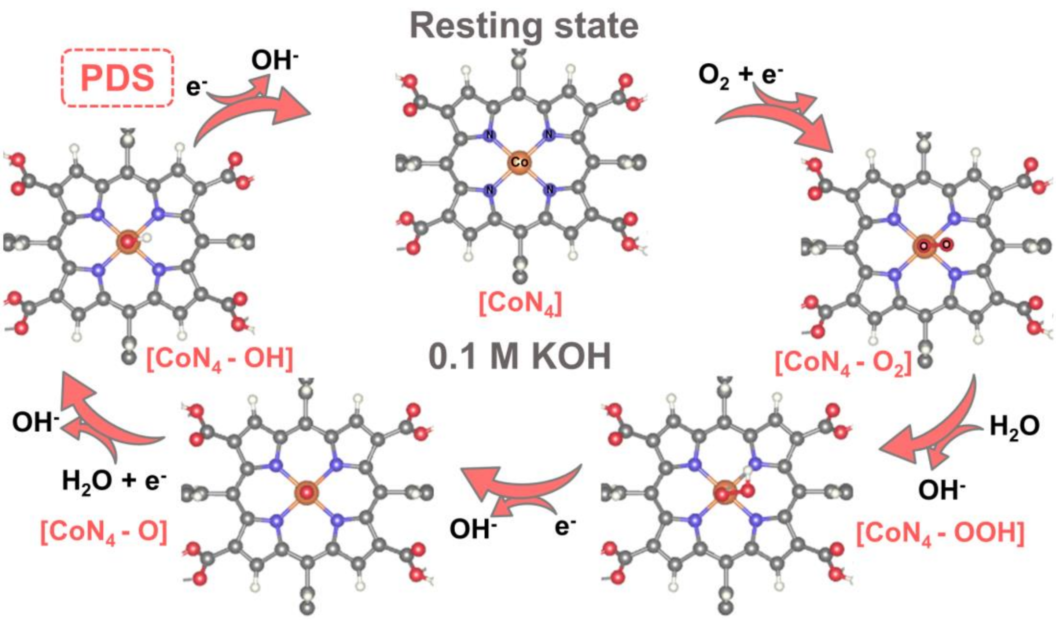

Supplementary Fig. 60. Schematic diagram of Co-N_4 site dynamic transfer for the electrocatalytic ORR over CoCOP-COOH@KB catalysts in 0.1 M KOH solution.

Supplementary Table 1. Mass contents of the metallic elements in CoCOP-X@KB catalysts from ICP-OES test.

Sample	Co contents (wt%)
CoCOP-COOH@KB	0.17
CoCOP-CH_3@KB	0.15
CoCOP-H@KB	0.14
CoCOP-$COCH_3$@KB	0.13
CoCOP-$COOCH_3$@KB	0.14
CoCOP-CN@KB	0.19

Supplementary Table 2. Summary of the Brunauer-Emmett-Teller (BET) surface area for CoCOP-X@KB and KB. The surface area was assessed by the Brunauer–Emmett–Teller (BET) method. The pore size distribution was evaluated by QSDFT method.

Sample	BET surface area (m^2 g^{-1})
CoCOP-COOH@KB	538.5
CoCOP-CH_3@KB	431.9
CoCOP-H@KB	465.3
CoCOP-$COCH_3$@KB	512.1
CoCOP-$COOCH_3$@KB	479.5
CoCOP-CN@KB	469.9
KB	1340.0

Supplementary Table 3. Comparison of SD_{mass} and TOF for CoCOP-COOH@KB, CoCOP-CH_3@KB, CoCOP-H@KB catalyst and reported catalysts in literatures.

Sample	SD (site g^{-1})	TOF (e $site^{-1}$ s^{-1})	Measurement method	Ref.
CoCOP-COOH@KB	7.27×10^{19}	4.60 @ 0.8 V	SECM	This work
CoCOP-CH_3@KB	2.38×10^{19}	0.60 @ 0.8 V	SECM	This work
CoCOP-H@KB	3.36×10^{19}	0.70 @ 0.8 V	SECM	This work
CoTAA-Ph(Cl)@GR	5.03×10^{19}	0.45 @ 0.8 V	SECM	[1]
PANI-Co	4.22×10^{19}	0.01 @ 0.8 V	CO adsorption	[2]
ZIF-Co	4.14×10^{19}	0.11 @ 0.8 V	CO adsorption	[2]
FeN_4	4.90×10^{19}	0.04 @ 0.8 V	Neutron activation analysis	[3]
$CFeN_2$	1.00×10^{19}	0.01 @ 0.8 V	Neutron activation analysis	[3]
Fe–$NC^{\Delta\text{-DCDA}}$	4.69×10^{19}	0.13 @ 0.85 V	Nitrite reduction	[4]
$Fe_{0.5}$NC-800	3.99×10^{19}	0.46 @ 0.9 V	CO adsorption	[5]
PAJ	2.00×10^{19}	0.7 @ 0.8 V	Nitrite reduction	[6]
CNRS	6.00×10^{19}	0.2 @ 0.8 V	Nitrite reduction	[6]

Supplementary Table 4. Summary of the EIS analysis. The numerical values and corresponding fit errors are obtained by fitting the raw data with the equivalent circuit modeling of EIS Nyquist curves for CoCOP-COOH@KB, CoCOP-CH_3@KB and CoCOP-H@KB. R_s, solution resistance. R_{ct}, charge transfer resistance. W, Warburg resistance.

Sample	R_s (Ω)	Error (R_s)	R_{ct} (Ω)	Error% (R_{ct})	W (Ω)	Error (W)
CoCOP-COOH@KB	22.8	0.3%	352.6	4.7%	33.4	4.6%
CoCOP-CH_3@KB	40.1	0.2%	353.3	3.7%	29.6	4.9%
CoCOP-H@KB	39.9	2.3%	389.8	4.4%	35.3	3.8%

References

1. Huang, B. Y. et al. Electron-donors-acceptors interaction enhancing electrocatalytic activity of metal-organic polymers for oxygen reduction. *Angew. Chem. Int. Ed.* **62**, e202306667 (2023).

2. Luo, F. et al. Kinetic diagnostics and synthetic design of platinum group metal-free electrocatalysts for the oxygen reduction reaction using reactivity maps and site utilization descriptors. *J. Am. Chem. Soc.* **144**, 13487-13498 (2022).

3. Koslowski, U. I., Abs-Wurmbach, I., Fiechter, S. & Bogdanoff, P. Nature of the catalytic centers of porphyrin-based electrocatalysts for the ORR: A correlation of kinetic current density with the site density of Fe−N_4 centers. *J. Phys. Chem. C* **112**, 15356-15366 (2008).

4. Mehmood, A. et al. High loading of single atomic iron sites in Fe–NC oxygen reduction catalysts for proton exchange membrane fuel cells. *Nat. Catal.* **5**, 311-323 (2022).

5. Luo, F. et al. Accurate evaluation of active-site density (SD) and turnover frequency (TOF) ofPGM-free metal–nitrogen-doped carbon (MNC) electrocatalysts using CO cryo adsorption. *ACS Catal.* **9**, 4841-4852 (2019).

6. Primbs, M. et al. Establishing reactivity descriptors for platinum group metal (PGM)-free Fe–N–C catalysts for PEM fuel cells. *Energ. Environ. Sci.* **13**, 2480-2500 (2020).
